# Identification and characterization of a human MORC2 DNA binding region that is required for gene silencing

**DOI:** 10.1093/nar/gkae1273

**Published:** 2024-12-31

**Authors:** Nikole L Fendler, Jimmy Ly, Luisa Welp, Dan Lu, Fabian Schulte, Henning Urlaub, Seychelle M Vos

**Affiliations:** Department of Biology, Massachusetts Institute of Technology, Building 68, 31 Ames St., Cambridge, MA 02139, USA; Department of Biology, Massachusetts Institute of Technology, Building 68, 31 Ames St., Cambridge, MA 02139, USA; Whitehead Institute for Biomedical Research, 455 Main St, Cambridge, MA 02139, USA; Bioanalytical Mass Spectrometry, Max Planck Institute for Multidisciplinary Sciences, Am Fassberg 11, 37077 Göttingen, Germany; Department of Clinical Chemistry, Bioanalytics Group, University Medical Center Göttingen, Robert-Koch-Straße 40 37075 Göttingen, Germany; Department of Systems Biology, Harvard Medical School, 210 Longwood Avenue, Boston, MA 02115, USA; Whitehead Institute for Biomedical Research, Quantitative Proteomics Core, 455 Main St, Cambridge, MA 02139, USA; Bioanalytical Mass Spectrometry, Max Planck Institute for Multidisciplinary Sciences, Am Fassberg 11, 37077 Göttingen, Germany; Department of Clinical Chemistry, Bioanalytics Group, University Medical Center Göttingen, Robert-Koch-Straße 40 37075 Göttingen, Germany; Department of Biology, Massachusetts Institute of Technology, Building 68, 31 Ames St., Cambridge, MA 02139, USA; Howard Hughes Medical Institute, 4000 Jones Bridge Rd, Chevy Chase, MD 20815, USA

## Abstract

The eukaryotic microrchidia (MORC) protein family are DNA gyrase, Hsp90, histidine kinase, MutL (GHKL)-type ATPases involved in gene expression regulation and chromatin compaction. The molecular mechanisms underlying these activities are incompletely understood. Here, we studied the full-length human MORC2 protein biochemically. We identified a DNA binding site in the C-terminus of the protein, and we observe that this region can be phosphorylated in cells. DNA binding by MORC2 reduces its ATPase activity and MORC2 can entrap multiple DNA substrates between its N-terminal GHKL and C-terminal coiled coil 3 dimerization domains. Finally, we observe that the MORC2 C-terminal DNA binding region is required for gene silencing in cells. Together, our data provide a model to understand how MORC2 engages with DNA substrates to mediate gene silencing.

## Graphical Abstract

**Graphical Abstract:**
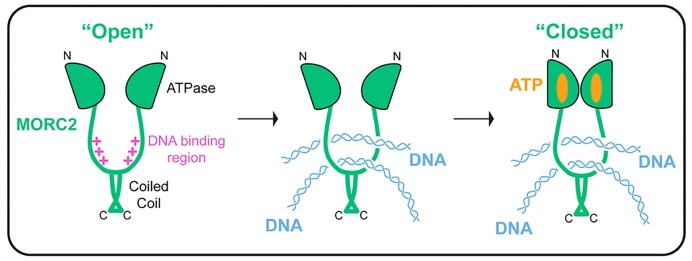

## Introduction

The microrchidia (MORC) protein family are DNA gyrase, Hsp90, histidine kinase and MutL (GHKL)-type ATPases involved in gene expression regulation (1). MORC proteins share a common architecture consisting of an N-terminal GHKL domain and a predicted C-terminal dimerization domain (2). Eukaryotic MORC proteins likely contribute to gene silencing by compacting chromatin. They are localized to specific genomic regions by post-translational modifications on themselves or histone proteins, or by association with protein complexes that associate with MORC proteins (3–8).

The exact molecular mechanisms underlying DNA binding and chromatin compaction by MORC family proteins are unknown. Members of the GHL subclass of the GHKL-type ATPase family, including type II topoisomerases, MutL and Hsp90, possess an N-terminal GHKL domain that dimerizes in the presence of ATP and a second C-terminal dimerization interface. GHL ATPases trap substrates in the lumen formed between the two dimerization interfaces (9–13). MORC proteins could similarly engage DNA between their two dimerization interfaces, which could lead to chromatin compaction if multiple DNA strands are engaged simultaneously (1,3,14–16). Indeed, *Caenorhabditis elegans* MORC-1 can topologically entrap DNA substrates, supporting this model (15). The regions of MORC1 that engage DNA, however, have not been identified.

Humans encode five MORC proteins (MORC1–4 and SMCHD1). MORC2, MORC3 and SMCHD1 are constitutively expressed in all cell types whereas MORC1 and MORC4 expression is limited to specific tissues (17, Human Protein Atlas). At a structural level, MORC1/MORC2 and MORC3/MORC4 share a similar domain architecture whereas SMCHD1 is structurally distinct from the other MORC family members (16,18–21). MORC proteins appear to regulate specific gene subsets. For example, MORC1 is responsible for silencing transposable elements in the male germline; MORC2 silences retrotransposons and genes with long exons; MORC3 silences virally derived DNAs and endogenous retroviruses; and SMCHD1 silences expression of the *Dux4* transcription factor (5,8,22–26). To date, the only MORC family protein that has been studied biochemically in its full-length form is *C. elegans* MORC-1 (15). All other biochemical and structural studies of MORC family proteins have been conducted with N-terminal truncations that lack the predicted C-terminal dimerization interface, and thus significant questions regarding MORC association with DNA remain unanswered (16,18–20).

**Figure 1.**
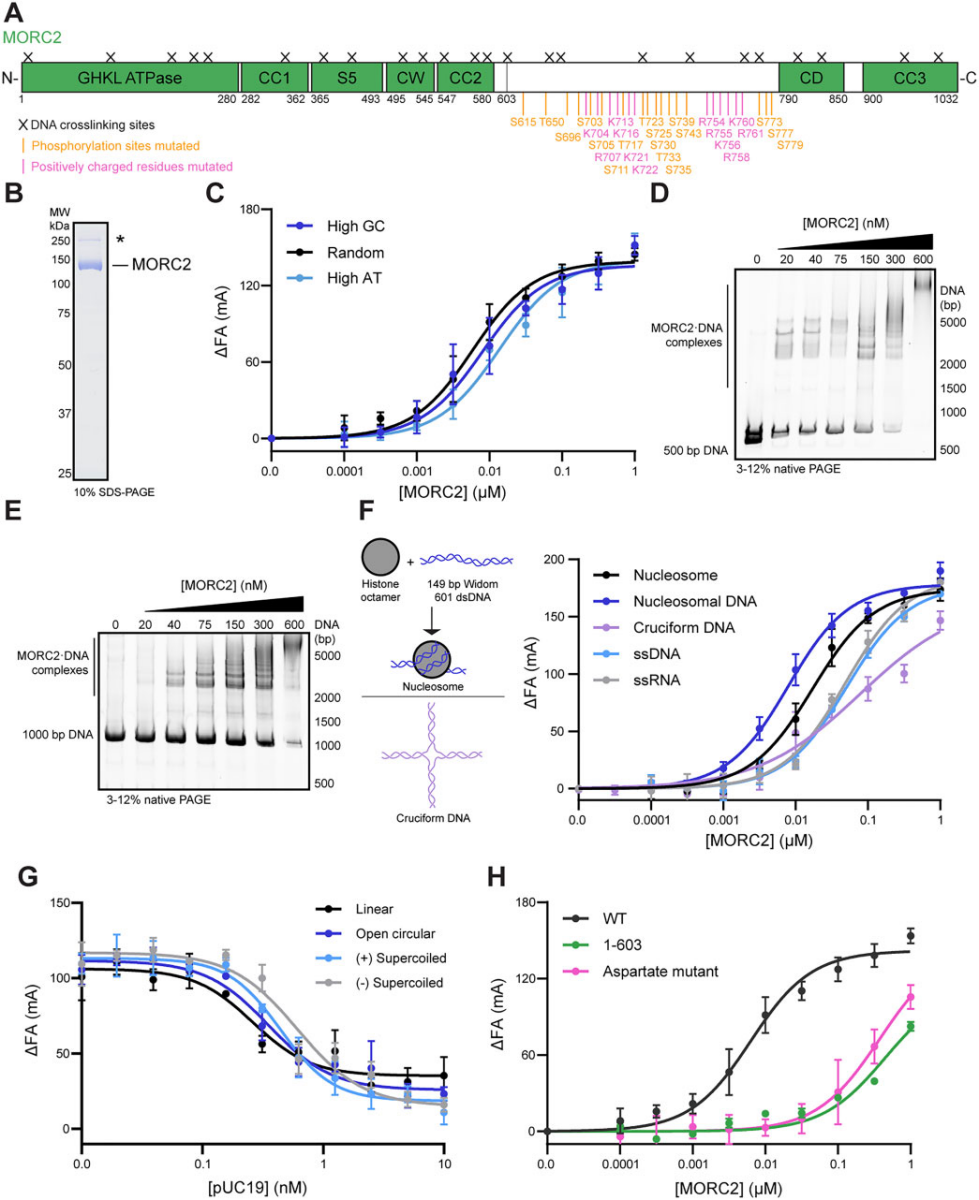
MORC2 preferentially associates with dsDNA via a C-terminal region. (**A**) Domain architecture of human MORC2. Structured domains are colored green. Coiled coil domains (CC), S5 transducer domain of the GHKL ATPase module (S5), a CW-type zinc finger domain (CW) and a predicted chromodomain (CD) are indicated. Purified MORC2 was cross-linked to DNA with 1 mM mechlorethamine for 30 min at 37°C or cross-linked to DNA by UV light at 254 nm for 10 min on ice, and samples were digested by trypsin. Resulting fragments were enriched by TiO_2_ and analyzed by mass spectrometry (‘Materials and methods’ section). Identified amino acid positions cross-linked to DNA are denoted by a black cross. Positively charged residues and phosphorylation sites mutated in this study are noted in pink and orange, respectively, beneath the primary structure. (**B**) SDS-PAGE gel of purified human MORC2 protein (3 μg) stained with Coomassie blue stain. Contaminant band marked with an asterisk (Supplementary Table S1). (**C**) MORC2 DNA binding to DNA sequences of different AT/GC content as assessed by fluorescence anisotropy. Dephosphorylated MORC2 was titrated and incubated with 1 nM 5′-FAM-labeled 35 base pair duplex DNA with a high GC (71% GC content), high AT (31% GC content) or a random sequence with 49% GC content (‘Materials and methods’ section). Binding curves were fit with a single site quadratic binding equation. Error bars correspond to the standard deviation between three replicate experiments. (**D**) MORC2 binding to a 500 bp DNA substrate assessed by gel shift. Increasing concentrations of dephosphorylated MORC2 was incubated with 20 nM of duplex DNA and resolved on a 3–12% gradient Native PAGE gel (‘Materials and methods’ section). (**E**) MORC2 binding to a 1000 bp DNA substrate assessed by gel shift. Increasing concentrations of dephosphorylated MORC2 was incubated with 20 nM of duplex DNA and resolved on a 3–12% gradient Native PAGE gel (‘Materials and methods’ section). (**F**) MORC2 nucleic acid binding as measured by fluorescence anisotropy. Dephosphorylated MORC2 was titrated and incubated with 1 nM 5′-FAM-labeled 149 bp Widom 601 dsDNA, nucleosome, cruciform DNA, 35 nucleotide ssRNA and 35 base pair ssDNA (‘Materials and methods’ section). Binding curves were fit as in 1C except for the cruciform DNA which was fit with a Hill equation. Error bars correspond to the standard deviation between three replicate experiments. (**G**) MORC2 association with DNAs of varying topologies. Dephosphorylated MORC2 (150 nM) was incubated with 5′-FAM-labeled 35 base pair duplex DNA (1 nM), and positively supercoiled, negatively supercoiled, or relaxed plasmid DNA was titrated into the reactions (‘Materials and methods’ section). Binding curves were fit using an inhibition curve with a variable response. Error bars correspond to the standard deviation between three replicate experiments. (**H**) Assessment of DNA binding by dephosphorylated wild-type, aspartate mutant and 1–603 MORC2. MORC2 constructs were titrated and incubated with a FAM-labeled 35 base pair duplex DNA (1 nM) (‘Materials and methods’ section). Binding curves were fit as in 1C. Error bars correspond to the standard deviation between three replicate experiments.

To study how MORC family proteins could associate with DNA in their full-length form, we have biochemically investigated full-length human MORC2. Human MORC2 is a 1032 amino acid protein containing an N-terminal GHKL ATPase domain bifurcated by a coiled coil, a CW-type zinc finger domain (CW), a predicted chromo-like domain (CD) and two predicted C-terminal coiled coil domains (Figure 1A). Human MORC2 represses actively transcribed double strand DNA viruses (27), retroelements, particularly evolutionarily young, long interspersed nuclear element-1 (LINE-1) (24,28–32), and intronless and long-exon containing genes (5,24). Gene silencing by MORC2 requires its ATPase activity and its three coiled coil domains (5). Although MORC2 contains two possible chromatin binding domains (CW and CD), MORC2’s CW domain does not bind histone tails (33) and the CD is dispensable for MORC2 silencing in cells (5). The MORC2 coiled coil 1 domain is the only region of MORC2 that has been shown to bind DNA (18). This interface lies on the periphery of the protein, and it is unclear how it would promote topological entrapment of DNA substrates.

Here we have identified a C-terminal region of human MORC2 that interacts robustly with DNA. The C-terminal DNA binding region is located within the predicted substrate binding lumen of the MORC2 dimer. Further, DNA binding diminishes MORC2 ATP hydrolysis activity. Finally, we show that MORC2 dimerization at its N- and C-termini results in the selective capture of circular DNA substrates, suggesting that it can encircle DNA and capture multiple DNA fragments simultaneously. Mutations to the MORC2 DNA binding region prevent capture of circular DNA substrates and ablate its gene silencing activity in cells. Together, our work identifies a conserved region of MORC2 that engages DNA substrates and provides a model for understanding how MORC2 and other MORC proteins contribute to gene silencing.

## Materials and methods

### Molecular cloning and protein expression

MORC2 (Uniprot ID: Q9Y6X9, isoform 1) was amplified from K562 cDNA and inserted into insect cell expression vector 438-B (Addgene plasmid: 55 219) (N-terminal 6x His (His_6_) tag followed by Tobacco etch virus (TEV) protease cleavage site and 438-C (Addgene plasmid: 55220) (N-terminal His_6_-Maltose binding protein [MBP] followed by TEV protease cleavage site) by ligation independent cloning (LIC) (34).

438-C MORC2 was used as a template for polymerase chain reaction (PCR) amplification to introduce mutations and generate MORC2 mutants N39A, E35A, aspartate DNA binding mutant (K704D, R707D, K713D, K716D, K721D, K722D, R754D, R755D, K756D, R758D, K760D, R761D), Subset N (K704D, R707D, K713D, K716D, K721D, K722D), Subset C (R754D, R755D, K756D, R758D, K760D, R761D), alanine DNA binding mutant (K704A, R707A, K713A, K716A, K721A, K722A, R754A, R755A, K756A, R758A, K760A, R761A), 1–603 and phosphodead (S615A, T650A, S696A, S703A, S705A, S711A, T717A, T723A, S725A, S730A, T733A, S735A, S739A, S743A, S773A, S777A, S779A) by sequence and ligation independent ligation independent cloning (SLIC). For phosphomimetic MORC2 (S615D, T650E, S696D, S703D, S705D, S711D, T717E, T723E, S725D, S730D, T733E, S735D, S739D, S743D, S773D, S777D, S779D), the region between amino acid 696 and 779 with phosphomimetic mutations was generated as a synthetic gene block (Integrated DNA Technologies) and cloned into MORC2 in 438-C by SLIC. For coiled coil 3, the region between amino acids 900 and 1032 was amplified by PCR and inserted into *Escherichia coli* expression vector 1B (N-terminal 6x His (His_6_) tag followed by TEV protease cleavage site) (Addgene plasmid: 29653).

For PiggyBac Enhanced Green Fluorescent Protein (EGFP)-tagged proteins, we first generated a tetracycline inducible EGFP plasmid containing the puromycin resistance marker (puromycin N-acetyltransferase). EGFP-TEV-S with multiple cloning sites followed by the bGH poly(A) signal was amplified by PCR and inserted downstream of the tetracycline-responsive promoter between cut sites NheI and SgfI in the HP138 puro plasmid (Addgene plasmid: 134246). MORC2 constructs were amplified by PCR and inserted downstream of TetO: EGFP-TEV-S between the KpnI and NotI restriction sites to generate tetracycline inducible EGFP-TEV-S-MORC2. These donor plasmids contained puromycin N-acetyltransferase (puromycin resistance), reverse tetracycline-controlled transactivator, and the tetracycline inducible EGFP-tagged transgene flanked by piggyBac inverted terminal repeats. To add the SV40 NLS (PKKKRKV) (35), an oligo duplex containing the SV40 NLS along with 20 bp homology arms was cloned upstream of EGFP-tagged MORC2 constructs using the NEB Gibson Assembly Master Mix (E2611L).

For spike in mRNA plasmids, Nano and Firefly luciferase flanked with the *Xenopus laevis* globin 5′- and 3′-UTR was cloned downstream of the T7 promoter using Gibson assembly. All clones were verified by Sanger sequencing and full plasmid sequencing (Primordium or Plasmidsaurus).

Purified plasmid DNA (0.3–0.8 μg) was electroporated into DH10αEMBacY cells to generate bacmids (36). Bacmids were prepared from positive clones by isopropanol precipitation and transfected into Sf9 cells grown in ESF921 (Expression Systems) with X-tremeGENE9 transfection reagent (Sigma) to generate V0 virus. V0 virus was harvested 48–72 h after transfection. V1 virus was produced by infecting 25 ml of Sf9 or Sf21 cells grown at 27°C, 300 rpm with 25–150 μl V0 virus. Cells were maintained at 1E6 cells/ml during virus production. V1 viruses were harvested 48–72 h after proliferation arrest and stored at 4°C. For protein expression, 600 ml of Hi5 cells grown in ESF921 medium were infected with 100–300 μl of V1 virus and grown for 60 h at 27°C. Cells were maintained at 1E6 cells/ml during expression. Cells were harvested by centrifugation (238 × *g*, 4°C for 30 min), resuspended in lysis buffer at 4°C (500 mM NaCl, 20 mM Na•HEPES pH 7.4, 10% glycerol (v/v), 5 mM β-mercaptoethanol (BME), 30 mM imidazole pH 8.0, 2 μM pepstatin A, 0.7 μM leupeptin, 1 mM phenylmethylsulfonyl fluoride, 2.8 mM benzamidine), snap frozen and stored at −80°C.

Coiled coil 3 was expressed in *E. coli* Bl21 (DE3) RIL cells. 2xYT media supplemented with 100 μg/ml kanamycin and 34 μg/ml chloramphenicol was inoculated an overnight culture. Cells were grown at 37°C, 160 rpm until reaching an optical density 0.4–0.5 at 600 nm. Protein expression was induced by adding 0.5 mM of isopropyl β-D-1-thiogalactopyranoside (IPTG) and cells were grown for an additional 4–5 h at 37°C. Cells were harvested by centrifugation at 4000 rpm, 4°C for 30 min, resuspended in lysis buffer at 4°C, snap frozen in liquid nitrogen and stored at −80°C.

### Protein purification

All steps were performed at 4°C unless otherwise specified. Frozen cell pellets were thawed in a room temperature water bath, and cells were lysed by sonication. Lysates were clarified by centrifugation. Clarified lysates were filtered through a 5 μm syringe filter, followed by additional filtration through 0.45 μm syringe filters. The filtered lysate was applied to a 5 ml HisTrap (Cytvia) column equilibrated in lysis buffer. The HisTrap column was washed with 10 CV of lysis buffer followed by 4 CV of high salt buffer (1 M NaCl, 20 mM Na•HEPES pH 7.4, 10% glycerol [v/v], 5 mM BME, 30 mM imidazole pH 8.0) followed by 4 CV of lysis buffer and 4 CV of low salt buffer (150 mM NaCl, 20 mM Na•HEPES pH 7.4, 10% glycerol [v/v], 5 mM BME, 30 mM imidazole pH 8.0). Tandem 5 ml HiTrap SP (Cytiva) and 5 ml HiTrap Q HP (Cytiva) columns equilibrated in low salt buffer were attached directly to the bottom of the HisTrap column. Protein was eluted from the HisTrap column with 9 CV of Nickel Elution buffer (150 mM NaCl, 20 mM Na•HEPES pH 7.4, 10% glycerol (v/v), 5 mM BME, 500 mM imidazole pH 8.0), after which the HiTrap S and HiTrap Q columns were detached from the HisTrap column. The HiTrap S and HiTrap Q columns were washed with 4 CV of low salt buffer and protein was eluted from the HiTrap S and HiTrap Q columns separately by a gradient of 0–100% low salt buffer to high salt buffer (flow rate of 1.5 ml/min for 30 min). Peak fractions were analyzed by 10% Tris-glycine SDS-PAGE followed by Coomassie staining. Fractions containing full-length protein were combined with 1.5 mg of His_6_-TEV protease (37) and dialyzed overnight in 7K MWCO SnakeSkin tubing (Fisher) against 1 L of dialysis buffer (500 mM NaCl, 20 mM Na•HEPES pH 7.4, 10% glycerol [v/v], 5 mM BME, 30 mM imidazole pH 8.0, 1 mM MnCl_2_). Dephosphorylated protein was generated by the addition of 0.4 mg of lambda protein phosphatase (prepared in house) at this step.

Protein was removed from the SnakeSkin tubing and applied to a 5 ml HisTrap column equilibrated in Lysis Buffer to remove uncleaved protein, TEV protease and the tag. Protein was concentrated in an Amicon (Millipore) 15 ml centrifugal concentrator 30K MWCO to 4–5 ml. The protein was applied to a S200 16/600 pg column (Cytvia) equilibrated in SEC buffer (500 mM NaCl, 20 mM Na•HEPES pH 7.4, 10% [v/v] glycerol and 1 mM tris(2-carboxyethyl)phosphine [TCEP]). Peak fractions were analyzed by 10% Tris-glycine SDS-PAGE followed by Coomassie staining. Peak fractions which contained full-length protein were pooled and concentrated in an Amicon (Millipore) 4 ml centrifugal concentrator 30K MWCO to 50–100 μM, aliquoted, flash frozen and stored at −80°C. Typical protein preparations yield 1–2 mg of wild-type MORC2 from 1.2 L of insect cell culture. MBP-tagged MORC2 was prepared as described above without the addition of TEV protease in the overnight dialysis and with a Superose 6 10/300 (Cytvia) column for the size exclusion.

### Coiled coil 3 purification

All steps were performed at 4°C unless otherwise specified. Frozen cell pellets were thawed in a room temperature water bath, and cells were lysed by sonication. Lysates were clarified by centrifugation (20 000 rpm, 30 min, A27 rotor). The lysate was applied to 2 × 5 ml HisTrap (Cytvia) columns equilibrated in lysis buffer. The HisTrap column was washed with 10 CV of lysis buffer followed by 4 CV of high salt buffer (1 M NaCl, 20 mM Na•HEPES pH 7.4, 10% glycerol [v/v], 5 mM BME, 30 mM imidazole pH 8.0) followed by 4 CV of lysis buffer. Protein was eluted from the HisTrap columns with a gradient from 0 to 100% Nickel Elution buffer (500 mM NaCl, 20 mM Na•HEPES pH 7.4, 10% glycerol (v/v), 5 mM BME, 500 mM imidazole pH 8.0) with a flow rate of 1.5 ml/min for 30 min. Peak fractions were analyzed by 15% Tris-glycine SDS-PAGE stained with Coomassie. Fractions containing full-length protein were combined with 1.5 mg of His_6_-TEV protease (37) and dialyzed overnight in 7K MWCO SnakeSkin tubing (Fisher) against 1L of Dialysis buffer (500 mM NaCl, 20 mM Na•HEPES pH 7.4, 10% glycerol (v/v), 5 mM BME, 30 mM imidazole pH 8.0).

Protein was removed from the SnakeSkin tubing and applied to a 5 ml HisTrap column equilibrated in Lysis Buffer to remove uncleaved protein, TEV protease and the tag. Protein was concentrated in an Amicon (Millipore) 15 ml centrifugal concentrator 3K MWCO to 4–5 ml. The protein was applied to a S75 16/600 pg column (Cytvia) equilibrated in SEC buffer (500 mM NaCl, 20 mM Na•HEPES pH 7.4, 10% (v/v) glycerol and 1 mM tris(2-carboxyethyl)phosphine (TCEP). Peak fractions were analyzed by 15% Tris-glycine SDS-PAGE followed by Coomassie staining. Peak fractions which contained full-length protein were pooled and concentrated in an Amicon (Millipore) 4 ml centrifugal concentrator 3K MWCO to 100–200 μM, aliquoted, flash frozen and stored at −80°C.

### Phospho-mass spectrometry analysis of heterologously overexpressed protein

Protein samples that were overexpressed in insect cells and subsequently purified were excised from Tris-glycine SDS-PAGE gels stained with Coomassie blue. Gel bands were divided into ∼2 mm squares and washed overnight in 50% methanol/ water. These were washed once more with 47.5/47.5/5% methanol/water/acetic acid for 2 h, dehydrated with acetonitrile and dried in a speed-vac. Reduction and alkylation of disulfide bonds was then carried out by the addition of 30 μl of 10 mM dithiothreitol (DTT) in 100 mM ammonium bicarbonate for 30 min to reduce disulfide bonds. The resulting free cysteine residues were subjected to an alkylation reaction by removal of the DTT solution and the addition of 100 mM iodoacetamide in 100 mM ammonium bicarbonate for 30 min to form carbamidomethyl cysteine. These were then washed with aliquots of acetonitrile, 100 mM ammonium bicarbonate and acetonitrile and dried in a speed-vac. The bands were enzymatically digested by the addition of 300 ng of trypsin in 50 mM ammonium bicarbonate to the dried gel pieces. These were allowed to digest overnight at 37°C with gentle shaking. The resulting peptides were extracted by the addition of 50 μl of 50 mM ammonium bicarbonate with gentle shaking for 10 min. The supernatant from this was collected in a 0.5 ml conical autosampler vial. Two subsequent additions of 47.5/47.5/5% acetonitrile/water/formic acid with gentle shaking for 10 min were performed with the supernatant added to the 0.5 ml of autosampler vial. Organic solvent was removed, and the volume was reduced to 20 μl using a speed vac for subsequent analyses.

The digestion extracts were analyzed by reversed phase high performance liquid chromatography (HPLC) using Waters NanoAcquity pumps and autosampler and a ThermoFisher Orbitrap Elite mass spectrometer using a nano flow configuration. A 20 mm × 180 μm column packed with 5 μm Symmetry C18 material (Waters) using a flow rate of 15 μl per min for 2 min to trap and wash peptides. These were then eluted onto the analytical column, which was self-packed with 3.6 μm Aeris C18 material (Phenomenex) in a fritted 20 cm × 75 μm fused silica tubing pulled to a 5 μm tip. The gradient was isocratic 1% A Buffer for 1 min at 250 nl per minute with increasing B buffer concentrations to 15% B at 58.5 min, 27% B at 89 min and 40% B at 103 min.

The mass spectrometer was operated in a dependent data acquisition mode where the 10 most abundant peptides detected in the Orbitrap using full scan mode with a resolution of 240 000 were subjected to daughter ion fragmentation in the linear ion trap. A running list of parent ions was tabulated to an exclusion list to increase the number of peptides analyzed throughout the chromatographic run. Peptides were identified from the MS data using Sequest (Thermo) algorithms to search for the sequence of human MORC2 and the *Trichoplusia ni* genome. A peptide threshold of 99.5% was used as cutoffs for identification of peptides and proteins with a two unique peptide cutoff. Phosphorylation modifications were identified computationally from peptide spectra without additional enrichment. Phosphorylated peptides were detected for MORC2 residues S268, S615, S696, T717, S725, S730, S739, S743, S777, S779, S856 and S1016 (Supplementary Figure S7A).

### DNA–protein cross-linking and mass spectrometry

#### MORC2-65mer DNA complex reconstitution, cross-linking and sample processing

3.3 μl MORC2 protein (132.3 μM, corresponding to 436.6 pmol, 51.6 μg protein) was incubated with 5.7 μl of 65mer DNA (100 μM, corresponding to 570 pmol) for 10 min on ice. The sequence of the 65 base pair DNA is as follows: 5′-/6-FAM-ATT CTC CAG GGC GGC CGC GTA TAG GGT CCA TCA GAA TTC GGA TGA ACT CGG TGT GAA GAA AGA TC-3′

1 μl of 20 mM AMP-PNP was added as well as 50 mM NaCl, 20 mM HEPES pH 7.4, 1 mM TCEP, 10% glycerol, 3 mM MgCl_2_, 0.025 mM ZnCl_2_ following incubation for 20 min, on ice, in a final volume of 20 μl. UV_254nm_ light cross-linking was performed using an in-house built cross-linking apparatus as previously described (38). Samples were irradiated for 10 min on ice. Mechlorethamine cross-linking was performed at a final concentration of 1 mM mechlorethamine (Sigma; 122564) for 30 min, 37°C, 300 rpm, followed by quenching with 50 mM Tris–HCl pH 7.5 and incubation for 5 min, room temperature, 300 rpm. UV- and mechlorethamine cross-linked samples were ethanol precipitated. After washing, pellets were dissolved in 4 M urea in 50 mM Tris–HCl pH 7.5. Samples were diluted to 1 M urea using 50 mM Tris–HCl pH 7.5. 1 mM MgCl_2_, 250 U Pierce™ Universal nuclease for Cell Lysis (Thermo Scientific™, 88700), 100 U nuclease P1 (NEB, M0660S) and 1 kU RNase T1 (Thermo Scientific™, EN0541) were added, following incubation for 3.75 h, 37°C, 300 rpm. Trypsin (Promega; V5111) was added at a 1:20 enzyme-to-protein ratio following incubation overnight, 37°C, 300 rpm. Sample cleanup was performed using C18 stage tips (Harvard Apparatus; 74–4107) according to manufacturer’s instructions (71). Briefly, columns were equilibrated with 100% ACN; 50% (v/v) ACN, 0.1% (v/v) formic acid; and three times 0.1% (v/v) formic acid. Samples were loaded twice and washed three times with 5% (v/v) ACN, 0.1% (v/v) formic acid. Elution was performed using 50% (v/v) ACN, 0.1% (v/v) formic acid and 80% (v/v) ACN, 0.1% (v/v) formic acid. Peptides were dried in a speed vac concentrator. Enrichment of cross-linked peptide-(oligo)nucleotides was performed as described (38) using Titansphere TiO_2_ 10 nm beads (GL Sciences; 5020–75010). After enrichment, samples were dried in a speed vac concentrator and subjected to LC-MS/MS measurements.

#### LC-MS/MS analysis

Enriched cross-linked peptides were injected onto a C18 PepMap100-trapping column (0.3 × 5 mm, 5 μm, Thermo Scientific™) connected to an in-house packed C18 analytical column (75 μm × 300 mm; Reprosil-Pur 120C18-AQ, 1.9 μm, Dr Maisch GmbH). Columns were equilibrated using 98% buffer A (0.1% [v/v] formic acid), 2% buffer B (80% [v/v] ACN, 0.1% [v/v] formic acid). Liquid chromatography was performed using an UltiMate-3000 RSLC nanosystem (Thermo Scientific™). Peptides were analyzed for 58 min using a linear gradient (5–45% buffer B (80% [v/v] ACN, 0.1% [v/v] formic acid) in 44 min) followed by a 4.8 min washing step at 90% of buffer B. Eluting peptides were analyzed on an Orbitrap Exploris 480 instrument (Thermo Scientific™). The following MS settings were used: MS1 scan range, *m/z* 350–1600; MS1 resolution, 120 000 FWHM; AGC target MS1, 1E6; maximum injection time MS1, 60 ms; isolation window, *m/z* 1.4; top 30 most abundant precursors were selected for fragmentation; collision energy (HCD), 28% or 30% (for UV- and mechlorethamine-cross-linked samples, respectively); charge states, 2 + to 6+; dynamic exclusion, 7 s; MS2 resolution, 30 000, AGC target MS2, 1e5; maximum injection time MS2, 120 ms. The lock mass option (*m/z* 445.120025) was used for internal calibration.

#### Data analysis

MS raw files were processed using the OpenMS framework for MS data analysis including NuXL tool (Urlaub group unpublished) and manual spectrum evaluation in TOPPView (39). For database search, the canonical protein sequence of MORC2 protein was used and default search settings with modifications, including variable modifications, oxidation (M), acetylation (N-term); enzyme, trypsin; maximum number of nucleotides attached, 2; and nucleotide adduct settings for UV or mechlorethamine cross-linking.

### Nucleosome preparation

A DNA consisting of 2 base pairs-Widom 601 sequence (145 base pairs)-2 base pairs was amplified by PCR using a 5′-/6-FAM forward primer (Sigma). The PCR products were pooled from four 96-well PCR plates (100 μl per well, 40 ml of total volume) and isopropanol precipitated. DNA was purified using a 1 ml Resource Q column (Cytiva) and eluted with a 22–34% NaCl gradient of TE Buffer (10 mM Tris pH 8.0, 2 M NaCl, 1 mM EDTA pH 8.0). Peak fractions were pooled, ethanol-precipitated and resuspended in water. The 2–601-2 DNA sequence is as follows: 5′-/6-FAM-ATA TCG ATG TAT ATA TCT GAC ACG TGC CTG GAG ACT AGG GAG TAA TCC CCT TGG CGG TTA AAA CGC GGG GGA CAG CGC GTA CGT GCG TTT AAG CGG TGC TAG AGC TGT CTA CGA CCA ATT GAG CGG CCT CGG CAC CGG GAT TCT GA T AT-3′

Nucleosomes were reconstituted essentially as described previously (40,41). Histone octamer and DNA were mixed at a 1:1 molar ratio in RB-High Buffer (20 mM HEPES, pH 7.4, 2 M KCl, 1 mM EDTA, pH 8.0 and 1 mM DTT), transferred to Slide-A-Lyzer Mini Dialysis Units 20K MWCO and gradient dialyzed against RB-low buffer (20 mM HEPES, pH 7.4, 30 mM KCl, 1 mM EDTA, pH 8.0 and 1 mM DTT) for 18 h at 4°C. The sample was further dialyzed against RB-low buffer for 2 h at 4 °C. The sample was centrifuged for 1 min at 11 000 rpm to collect precipitate. Nucleosome concentration was quantified by absorbance at 280 nm. The molar extinction coefficient of the nucleosome was obtained by summing the molar extinction coefficients of the octamer and the DNA components at 280 nm.

### Cruciform preparation

Cruciform DNA was prepared as described previously (42). Single-stranded DNA oligos were obtained from Sigma-Aldrich and dissolved in water to 1 nmol/μl. Sequences of the DNA oligos used are detailed in Table 1. 2 nmol of single-stranded DNA oligos 1, 2 and 4 were combined with 1.8 nmol of DNA oligo 3 in TMS buffer (100 mM NaCl, 10 mM Tris–HCl pH 7.5, 10 mM MgCl_2_) to a final volume of 30 μl. The reaction was incubated for 5 min at 95°C and then cooled by 1°C/min until the sample reached 4°C. To verify completion of the cruciform annealing, 3 μl of the cruciform reaction was mixed with 3 μl of 50% sucrose and applied to a 10% 0.2× TBE native PAGE gel run at 150 V for 1 h. The gel was stained with SYBR gold (Supplementary Figure S1D).

**Table 1.** DNA oligo sequences used for cruciform preparation

1	5′-CCC TAT AAC CCC TGC ATT GAA TTC CAG TCT GAT AA-3′
2	5′-GTA GTC GTG ATA GGT GCA GGG GTT ATA GGG-3′
3	5′/6-FAM-AAC AGT AGC TCT TAT TCG AGC TCG CGC CCT ATC ACG ACT A-3′
4	5′-TTT ATC AGA CTG GAA TTC AAG CGC GAG CTC GAA TAA GAG CTA CTG T-3′

### Preparation of relaxed and positively supercoiled plasmid substrates

pUC19 plasmid DNA was prepared by maxiprep following the manufacturer’s instructions (Qiagen). Relaxed pUC19 was prepared by incubating 50 μg pUC19 with Nt.BspQI at 50°C for 8 h. The reaction was heat inactivated at 80°C for 20 min. Nicked plasmid was ligated by adding T4 ligase and ATP and incubated overnight at room temperature. Relaxed plasmid DNA was purified by phenol chloroform extraction and precipitated with isopropanol. Positively supercoiled pUC19 was prepared by incubating 50 μg pUC19 with 1 μM *A. fulgidus* reverse gyrase (purified in house) (43) and 2 mM ATP in reverse gyrase buffer (10 mM NaCl, 10 mM MgCl_2_, 250 mM Tris–HCl pH 7.9) at 95°C for 30 min. The reaction was quenched with 1% (w/v) SDS and 10 mM EDTA pH 8.0 (final concentrations). Positively supercoiled pUC19 was purified by phenol chloroform extraction, precipitated with isopropanol, and resuspended in water.

### Electromobility shift assay

500 and 1000 bp DNA substrates were amplified by PCR from pUC19. The sequences of the primers used are detailed in Table 2. The PCR products were pooled from two 96-well PCR plates (100 μl per well, 20 ml total volume) and isopropanol precipitated. DNA was purified using a 1 ml Resource Q column (Cytiva) and eluted with a 22–34% NaCl gradient in TE Buffer (10 mM Tris pH 8.0, gradient from 0 to 2 M NaCl, 1 mM EDTA pH 8.0). Peak fractions were pooled, ethanol-precipitated and resuspended in water.

**Table 2.** DNA primer sequences used to prepare electromobility shift assay substrates

500 bp DNA Forward	5′ -TGC CGC TTA CCG GAT ACC-3′
500 bp DNA Reverse	5′ -TTC GTT CCA CTG AGC GTC AG-3′
1000 bp DNA Forward	5′-TGC CGC TTA CCG GAT ACC-3′
1000 bp DNA Reverse	5′-CGT TGG GAA CCG GAG C-3′

MORC2 protein was diluted in half in Half Dilution buffer (20 mM Na•HEPES pH 7.4, 10% [v/v] glycerol and 1 mM TCEP) and then serially diluted in half log steps in Protein Dilution buffer (250 mM NaCl, 20 mM Na•HEPES pH 7.4, 10% [v/v] glycerol and 1 mM TCEP). 500 or 1000 bp DNA (6 μl, 20 nM final concentration) was mixed with MORC2 dilutions (4 μl, 15–600 nM final concentrations) on ice and incubated for 10 min. The assay was brought to a final volume of 20 μl and incubated at room temperature for 30 min (final conditions: 50 mM NaCl, 3 mM MgCl_2_, 20 mM Na-HEPES pH 7.4, 1 mM TCEP, 10% [v/v] glycerol, 0.025 mM ZnCl_2_ and 50 μg/ml BSA). Samples were supplemented with 5 μl of 50% glycerol, and 10 μl was loaded into a 3–12% gradient Native PAGE gel (Invitrogen). Gels were run at 100 V for 4 h in 1X NativePAGE running buffer (Invitrogen) and stained with SYBR gold.

### Fluorescence anisotropy

5′-/6-FAM labeled double-stranded DNA (dsDNA) oligos were obtained from IDT. 5′-/6-FAM labeled single-stranded DNA (ssDNA) and single-stranded RNA (ssRNA) were obtained from Sigma-Aldrich and dissolved in water to 100 μM. Sequences of nucleic acid substrates used are detailed in Table 3.

**Table 3.** Oligo sequences used for fluorescence anisotropy experiments

Random 35mer dsDNA	5′-/6-FAM-ATA GGG TCC ATC AGA ATT CGG ATG AAC TCG GTG TG-3′
High AT 35mer dsDNA	5′-/6-FAM-CAT CAT CAA AGA CCA AAA GTA GAT AAA ACC ACA AA-3′
High GC 35mer dsDNA	5′-/6-FAM-TGT GTG CGC ACC GTG CGC GAG CCG AAG CAG GGC GA-3′
Random 35mer ssDNA	5′-/6-FAM-ATA GGG TCC ATC AGA ATT CGG ATG AAC TCG GTG TG-3′
Random 35mer ssRNA	5′-/6-FAM-AUA GGG UCC AUC AGA AUU CGG AUG AAC UCG GUG UG-3′
Random 25mer dsDNA	5′-/6-FAM-TCC ATC AGA ATT CGG ATG AAC TCG G-3′
Random 45mer dsDNA	5′-/6-FAM- GCC GCG TAT AGG GTC CAT CAG AAT TCG GAT GAA CTC GGT GTG AAG-3′
Random 65mer dsDNA	5′-/6-FAM- ATT CTC CAG GGC GGC CGC GTA TAG GGT CCA TCA GAA TTC GGA TGA ACT CGG TGT GAA GAA AGA TC-3′

MORC2 protein was diluted in half in Half Dilution buffer and subsequently serially diluted in half log steps in Protein Dilution buffer. Nucleic acid (5 μl, 1 nM final concentration) was mixed with MORC2 dilutions (5 μl, 1–0.001 μM final concentrations) on ice and incubated for 10 min. The assay was brought to a final volume of 25 μl and incubated in the dark at room temperature for 20 min (final conditions: 50 mM NaCl, 3 mM MgCl_2_, 20 mM Na-HEPES pH 7.4, 1 mM TCEP, 10% [v/v] glycerol, 0.025 mM ZnCl_2_ and 50 μg/ml BSA). 18 μl of each solution was transferred to a Greiner 384 Flat Black Bottom plate.

For competition experiments, MORC2 protein was initially diluted in half in Half Dilution buffer and then further diluted to 750 nM in Protein Dilution buffer. 35mer dsDNA (5 μl, 1 nM final concentration) was mixed with MORC2 (5 μl, 150 nM final concentration) on ice and incubated for 10 min. pUC19 plasmid DNA was serially diluted in water and was added to the MORC2·DNA mixture (10 μl, 0–20 nM final concentrations). The assay was brought to a final volume of 25 μl and incubated in the dark at room temperature for 20 min (final conditions: 50 mM NaCl, 3 mM MgCl_2_, 20 mM Na-HEPES pH 7.4, 1 mM TCEP, 10% [v/v] glycerol, 0.025 mM ZnCl_2_ and 50 μg/ml BSA). 18 μl of each solution was transferred to a Greiner 384 Flat Black Bottom plate.

Fluorescence anisotropy was measured at room temperature with a Tecan SPARK plate reader with an excitation wavelength of 470 nm (±5 nm), an emission wavelength of 518 nm (±20 nm), a gain of 150 and a Z-height of 26 050 μm. Experimental measurements with protein were normalized to buffer with nucleic acids alone. All experiments were done in triplicate. All binding curves, except for cruciform binding and competition binding, were fit with a single site quadratic binding equation:


\begin{eqnarray*}y = {{b}_{{\rm max}}}\frac{{\left( {\left[ {\mathrm{L}} \right] + \left[ {\mathrm{P}} \right] + {{K}_{{\rm d},\ {\rm app}}}} \right) - \sqrt {{{{\left( {\left[ L \right] + \left[ P \right] + {{K}_{{\rm d},\ {\rm app}}}} \right)}}^2} - 4\left[ L \right]\left[ P \right]} }}{{2\left[ L \right]}}\end{eqnarray*}

where *B*_max_ is the maximum specific binding, *L* is the concentration of nucleic acid, *P* is the concentration of MORC2, and *K*_d, app_ is the apparent dissociation constant.

Cruciform binding was fit with a Hill equation:


\begin{eqnarray*}y = \ \frac{{{{b}_{{\rm max}}}{{{\left( x \right)}}^h}}}{{{{{({{K}_{{\rm d},\ {\rm app}}})}}^h} + \ {{x}^h}}}\end{eqnarray*}

where *B*_max_ is the maximum specific binding, *h* is the Hill slope, *x* is the concentration of MORC2, and *K*_d, app_ is the apparent dissociation constant.

Competition binding experiments were fit with an inhibition curve with a variable response:


\begin{eqnarray*}y = \ \frac{{{\rm Min} + \left( {{\rm Max} - {\rm Min}} \right)}}{{1 + {{{\left( {\frac{{IC50}}{x}} \right)}}^h}}}\end{eqnarray*}

where Min is the minimum anisotropy value, Max is the maximum anisotropy value, *h* is the Hill slope, *x* is the concentration of plasmid DNA, and IC50 is the inhibition constant.

Curve fitting was performed in GraphPad Prism (10.0.3).

### Malachite green ATPase assay

MORC2 ATP hydrolysis activity was measured using a malachite green assay. MORC2 protein was initially diluted in half in Half Dilution buffer and then diluted to 5 μM in Protein Dilution buffer. MORC2 (10 μl, 1 μM final concentration) was mixed with water or 35 base pair random dsDNA substrate (10 μl, 2 μM final concentration or as indicated) on ice for 10 min. The assay was brought to a volume of 45 μl and incubated at 37°C for 5 min (final conditions: 50 mM NaCl, 3 mM MgCl_2_, 20 mM Na-HEPES pH 7.4, 1 mM TCEP, 10% [v/v] glycerol, 0.025 mM ZnCl_2_, 2 mM phosphoenol pyruvate, 4 U Pyruvate Kinase/ Lactate Dehydrogenase, and 50 μg/ml BSA). Ultrapure ATP (Jena Bioscience) was added to the reaction (5 μl, 1 mM final concentration or as indicated). After incubating the reactions for 45 min at 37°C, reactions were quenched with EDTA pH 8.0 (20 mM final concentration) and moved to ice. For ATPase measurements where ATP was titrated, reactions were prepared as described above except with 10 mM MgCl_2_ final concentration to provide enough Mg^2+^ ions to coordinate the excess ATP in the reaction. For each experiment, a titration of sodium phosphate from 5 to 500 μM in the final reaction conditions was made to generate a standard curve.

Sulfuric acid (320 mM final concentration) was added to precipitate protein on ice for 10 min. The malachite green detection mixture was prepared on ice (1 ml 0.12% [w/v] malachite green, 330 μl 14% [w/v] ammonium molybdate, 20 μl 11% [v/v] Tween-20). Quenched and precipitated samples were centrifuged at 15 000 rpm for 1 min. 50 μl of samples were transferred to a Greiner 384 Flat Clear Bottom plate. 25 μl of malachite green detection mixture was added for a 2-min incubation in the dark, and then 7.5 μl 34% (w/v) sodium citrate was added for an additional 28-min incubation in the dark. Absorbance at 620 nm was measured in a Tecan SPARK plate reader at room temperature. Experimental measurements with protein were normalized to a buffer control with substrate alone. All experiments were done in triplicate. The standard curve was used to convert absorbance values to the concentration of free phosphate. The concentration of free phosphate was assumed to equal the amount of ATP hydrolyzed by MORC2. ATP hydrolysis rates are reported as μM ATP hydrolyzed per μM MORC2 protein per minute. The ATP titration data are fit to the Michaelis–Menten model:


\begin{eqnarray*}V = \frac{{\left( {{{V}_{{\rm max}}}\ \times \left[ S \right]} \right)}}{{{{K}_{\rm m}} + \left[ S \right]}}\end{eqnarray*}

where *V* is the reaction rate, *V*_max_ is the maximum rate, [*S*] is the total starting substrate concentration, and *K*_m_ is the Michaelis–Menten constant. Curve fitting was performed in GraphPad Prism (10.0.3). The DNA titration data were fit to an inhibition curve:


\begin{eqnarray*}R = {\rm Min} + \frac{{\left( {{\rm Max} - {\rm Min}} \right)}}{{1 + \ \frac{{\left[ I \right]}}{{IC50}}}}\end{eqnarray*}

where *R* is the rate, Min is the minimum rate, Max is the maximum rate, [*I*] is the inhibitor concentration, and IC50 is the inhibition constant. Curve fitting was performed in GraphPad Prism (10.0.3).

### Size-exclusion chromatography (SEC)-coupled to multi-angle light scattering (MALS)

Size-exclusion chromatography (SEC)-multi-angle light scattering (MALS) analysis of MORC2 constructs was performed using 100 μl of protein in final conditions: 3 mM MgCl_2_, 20 mM Na-HEPES pH 7.4, 0.5 mM TCEP, 1.5% (v/v) glycerol and 0.025 mM ZnCl_2_. For full-length MORC2, 2 mg/ml of MORC2 was prepared in 150 mM NaCl with or without the addition of 1 mM AMP-PNP. For 1–603 MORC2, 1 mg/ml of 1–603 MORC2 was prepared in 50 mM NaCl in the presence or absence of 1 mM AMP-PNP. Samples were analyzed on an analytical 030 column (Wyatt) run at a flow rate of 0.5 ml/min. 1.5 mg/ml of coiled coil 3 protein was prepared in 150 mM NaCl and was analyzed on an analytical 010 column (Wyatt). Light scattering analysis was performed in ASTRA (Wyatt), using band broadening parameters obtained from a BSA standard in identical running conditions. MALS data were used to fit the average molar mass across the peak of interest, reported to the nearest kDa.

### Plasmid capture assay

Plasmid substrates, pUC19 and pBlueScript, were prepared by maxiprep following the manufacturer’s instructions (Qiagen). Linear pUC19 substrate was prepared by incubating 50 μg of pUC19 with EcoRI for 6 h at 37°C. Linear pUC19 was purified by phenol chloroform extraction and precipitated with isopropanol.

MBP-tagged MORC2 was diluted in half in Half Dilution buffer and then further diluted in Protein Dilution buffer. Diluted MORC2 (600 nM final, monomer concentration) was combined with circular or linear substrate (100 nM final) and incubated at room temperature for 20 min. The assay was brought to 15 μl final volume in the presence or absence of 1 mM AMP-PNP and incubated at room temperature for 30 min (final conditions: 50 mM NaCl, 3 mM MgCl_2_, 20 mM Na-HEPES pH 7.4, 1 mM TCEP, 10% [v/v] glycerol, 0.025 mM ZnCl_2_ and 50 μg/ml BSA). 50 μl amylose resin was washed with water and equilibrated in reaction buffer (50 mM NaCl, 3 mM MgCl_2_, 20 mM Na-HEPES pH 7.4, 1 mM TCEP, 10% [v/v] glycerol, 0.025 mM ZnCl_2_ and 50 μg/ml BSA). 1 μl of the reaction was saved as an input sample and the remaining 14 μl of the reaction was applied to the equilibrated amylose resin. The reaction mixture was incubated with the amylose resin for 30 min at room temperature shaking at 800 rpm. The mixture was washed twice with 250 μl of either reaction buffer or a high salt buffer (400 mM NaCl, 3 mM MgCl_2_, 20 mM Na-HEPES pH 7.4, 1 mM TCEP, 10% [v/v] glycerol and 0.025 mM ZnCl_2_). Samples with AMP-PNP were washed with buffer supplemented with 1 mM AMP-PNP. Protein was eluted from the resin with 20 μl Maltose Elution buffer (50 mM NaCl, 3 mM MgCl_2_, 20 mM Na-HEPES pH 7.4, 1 mM TCEP, 10% [v/v] glycerol, 0.025 mM ZnCl_2_, 50 μg/ml BSA and 100 mM maltose). Reactions were quenched with EDTA (10 mM final). Input samples were diluted to 10 μl with reaction buffer. Protein in all samples and input samples was digested by proteinase K. All samples were supplemented with 10% sucrose and visualized on 1% (w/v) 1x TAE (50 mM Tris–HCl, pH 7.9, 1 mM EDTA, pH 8.0, 40 mM sodium acetate) agarose gels with 1:10 000 SYBR Safe stain (Apex) run at 100 V for 50 min. Gels were imaged on a BioRad Gel Dock imager with a 1.5 s exposure.

Gel images were quantified in ImageJ (1.52A). A 60 × 40 pixel box was drawn around the input or retained DNA band for each lane and the integrated density inside the box was measured. For each lane, a 60 × 40 pixel box was drawn below the DNA band and the integrated density inside the box was measured as the background. The reported values are the difference between the integrated density measurements of the DNA and the background for each lane.

### Dual DNA capture assay

DNA bead stocks were prepared essentially as described before (44). pUC19 DNA was amplified by PCR with the following primers: 5′-biotin-CGG TGA AAA CCT CTG ACA CAT G-3′ and 5′-biotin-TCA TCA CCG AAA CGC GC-3′. The desired product was separated from PCR byproducts and primers on a 1 ml Resource Q column (Cytiva). Samples were loaded on the column in TE Zero buffer (10 mM Tris–HCl, pH 8.0, 1 mM EDTA, pH 8.0), washed with 28% TE High buffer (10 mM Tris–HCl, pH 8.0, 1 mM EDTA, pH 8.0, 2M NaCl) and eluted by a gradient from 28% to 34% TE High buffer. Fractions containing the desired product were pooled and DNA was isopropanol precipitated. Dynabeads M-280 Streptavidin beads (Invitrogen) were washed three times in DNA Binding buffer (10 mM HEPES, pH 7.4, 2M NaCl, 1 mM EDTA, pH 8.0). 2.5 μg of DNA per 100 μl of beads was added and the mixture was diluted in half with water. Samples were incubated overnight at room temperature with gentle rotation. Beads were washed three times in reaction buffer and stored at 4°C. For the three DNA substrate experiments, pBlueScript plasmid with a Widom 601 sequence insert (‘pBlueScript-601’) was prepared by maxiprep following the manufacturer’s instructions (Qiagen).

MORC2 was diluted in half with Half Dilution buffer and then further diluted in Protein Dilution buffer. Diluted MORC2 (600 nM final) was combined with 20 μl DNA beads and incubated at room temperature for 20 min with shaking at 800 rpm in the presence or absence of 1 mM AMP-PNP. Either pBlueScript (200 nM final) or pBlueScript and pBlueScript-601 (100 nM each, 200 nM combined plasmid DNA final) was added, and the mixture was incubated at room temperature for 30 min, shaking. For AMP-PNP second samples, 1 mM AMP-PNP was added after incubation with plasmid DNA, and the samples were incubated at room temperature for 30 min, shaking. Samples were washed twice with 250 μl of either reaction buffer or high salt buffer (400 mM NaCl, 3 mM MgCl_2_, 20 mM Na-HEPES pH 7.4, 1 mM TCEP, 10% [v/v] glycerol and 0.025 mM ZnCl_2_). Samples with AMP-PNP were washed with buffer supplemented with 1 mM AMP-PNP. Beads were resuspended in 1X CutSmart Buffer (New England Biolabs), and DNA was digested with 20 U ScaI-HF and 20 U Sbf-HF for 1 h at 37°C. Supernatants were removed and protein was digested with proteinase K for 1 h at 37°C. All samples were supplemented with 10% sucrose and visualized on 1% (w/v) 1× TAE (50 mM Tris–HCl, pH 7.9, 1 mM EDTA, pH 8.0, 40 mM sodium acetate) agarose gels with 1:10 000 SYBR Safe stain (Apex) run at 100 V for 50 min. Gels were imaged on a BioRad Gel Dock imager with a 1.5 s exposure.

Gel images were quantified in ImageJ (1.52A). A 60 × 30 pixel box was drawn around the input bead DNA band and the retained second plasmid DNA band for each lane and the integrated density inside the box was measured. For each lane, a 60 × 30 pixel box was drawn below the input bead DNA band and the integrated density inside the box was measured as the background. The reported values are the ratio of the difference between the integrated density measurements of the retained second plasmid DNA and the background for each lane and the difference between the integrated density measurements of the input bead DNA and the background for each lane.

### Circular dichroism

MORC2 protein samples were dialyzed overnight at 4°C with stirring against CD Dialysis Buffer (100 mM NaCl, 10 mM HEPES, pH 7.4). Samples were retrieved and diluted to 0.1 mg/ml in CD Dialysis Buffer. CD spectra were taken at 25°C with a JASCO model J-1500 Circular Dichroism Spectrophotometer in a 1-mm pathlength quartz cuvette. Data were obtained from 250 to 200 nm, at 0.5 nm intervals. Each point is averaged over 4 s, and each read was performed three times. Reported values are the difference between the protein sample and the buffer alone control.

### NLS stradmus analysis

NLS Stradmus tool for nuclear localization signal prediction was described previously (45). For MORC2, a 2-state static Hidden Markov Model was used, and sequences were selected above a 0.4 posterior threshold cutoff.

### AlphaFold multimer

The AlphaFold Multimer dimer structure of full-length MORC2 was predicted using the COSMIC2 server (46). The structure was visualized in PyMOL (2.5.2).

### Multiple sequence alignment

Primary sequences of MORC2 proteins from various organisms were obtained from Uniprot (IDs: Q9Y6X9, K7D393, F1RPD9, M3W1K4, A0A8C0I0T3, Q69ZX6, D4A2C4, A0A1L8HZT1, A0A670ZP14, Q68EG7). Sequences were aligned using MAFFT (version 7) and visualized in Jalview (2.11.3.0).

### Cell culture conditions

MORC2 knockout HeLa cells were generated by Ubigene Biosciences using a CRISPR gene editing strategy. Briefly, HeLa cells were electroporated with Cas9 protein and gRNAs flanking exon 5 of the MORC2 gene, followed by single clone selection. Sequences of the gRNAs are detailed in Table 4 with the PAM sequence italicized.

**Table 4.** Guide RNA sequences used to generate MORC2 knockout cells

gRNA -1	5′-GTA TAT TAG AAG CTT GTC AC *AGG*-3′
gRNA -2	5′-ACA GTG ATA GAG GTA CCA AC *AGG-*3′

To genotype MORC2 knockouts, cells were lysed in DirectPCR lysis reagent (Viagen, 301-C) with proteinase K (0.2 μg/ml) overnight at 55°C then proteinase K was inactivated at 85°C for 90 min. Clones were screened by PCR amplification of a region of the MORC2 gene around exon 5 such that removal of exon 5 would result in a shorter PCR product. The resulting PCR product was also validated by Sanger sequencing (Quintara). Sequences of the primers used for PCR amplification are detailed in Table 5.

**Table 5.** PCR primers used to verify MORC2 knockout cells

PCR-Forward	5′-GAC GAG AGG ACC TTC GAG G-3′
PCR-Reverse	5′-AGG TAG GTC CCT GCT ATA AGG-3′

HeLa cell lines (a gift from the lab of Iain Cheeseman) and the MORC2 knockout HeLa cell lines were cultured in DMEM supplemented with 10% tetracycline-free fetal bovine serum (FBS), 2 mM L-glutamine and 100 U/ml penicillin/streptomycin at 37°C with 5% CO_2_. The piggyBac transposon system (47) was used to introduce EGFP-tagged MORC2 constructs to wild-type or MORC2 knockout HeLa cells. Cells were plated in six-well plates. To generate stable tetracycline inducible cell lines, 1 μg donor plasmid containing the tetracycline inducible transgene was co-transfected with 400 ng piggyBac transposase plasmid (HP137, a gift from the lab of Rudolf Jaenisch) using lipofectamine 2000. Cells were grown at 37°C for 1 day, then the media was exchanged. Forty-eight hours post transfection, cells were moved into a 10 cm^2^ plate and selected for puromycin resistance using puromycin 0.35 μg/ ml for 5 days. Cell lines were frozen at ∼1 million cells/ml in Dulbecco's Modified Eagle Medium (DMEM) supplemented with 25% FBS and 10% Dimethyl sulfoxide (DMSO) and stored in liquid nitrogen.

### Protein extraction and western blots

Pellets of ∼5 million HeLa cells were thawed on ice and resuspended in lysis buffer (2X TBS, 1% Triton X-100, 1X HALT protease and phosphatase inhibitor cocktail). Protein concentrations were quantified using BCA Protein Assay Kit (Thermo Scientific) following the manufacturer’s protocol. 50 μg of total proteins were loaded into each lane of a 4–12% Bis-Tris gradient gel (NuPAGE) and transferred to 0.45 μm PVDF membranes (GE Healthcare). Membranes were blocked with 5% BSA in TBST (Tris buffered saline pH 8.0, 0.1% Tween 20, Thermo Scientific) for 1 h at room temperature and incubated with primary antibody overnight at 4°C. Membranes were washed with TBST and incubated with secondary HRP conjugate antibodies for 1 h at room temperature, followed by washing with TBST and development using a chemiluminescence substrate (Thermo Fisher #34 580). Membranes were imaged using a Bio-rad Chemidoc. Primary antibodies used: β-ACT (Sigma-Aldrich #A5316, 1:10 000 dilution in 5% BSA in TBST), MORC2 (Bethyl Laboratories #A300-149A, 1:1000 dilution in 5% BSA in TBST), α-tubulin (Invitrogen #32–2500, 1:5000 dilution in 5% BSA in TBST), Lamin A/C (CST #2032, 1:1000 dilution in 5% BSA in TBST) and Histone H3 (EpiCypher #13–0001, 1:2500 dilution in 5% BSA in TBST). Secondary antibodies used: anti-mouse IgG (Invitrogen#A-865, 1:10 000 dilution in 5% BSA in TBST), anti-rabbit IgG (Invitrogen #31460, 1:10 000 dilution in 5% BSA in TBST).

### Sub-cellular fractionation and immunoprecipitation

Approximately 100 million HeLa cells were used for each fractionation experiment as described previously (48). All steps were performed at 4°C. Cells were collected by scraping and lysed in 750 μl E1 buffer (50 mM HEPES pH 7.5, 140 mM NaCl, 1 mM EDTA pH 8.0, 10% [v/v] glycerol, 0.5% NP-40, 0.25% Triton X-100, 1 mM DTT, 2 μM pepstatin A, 0.7 μM leupeptin, 1 mM phenylmethylsulfonyl fluoride, 2.8 mM benzamidine) supplemented with phosphatase inhibitor (Roche PhosSTOP #12352200). Lysates were centrifuged at 1100 × *g* for 2 min. Supernatants were collected as cytosolic fractions. Pellets were washed once with 1 ml E1 buffer and resuspended in 750 μl E2 buffer (10 mM Tris–HCl pH 8.0, 200 mM NaCl, 1 mM EDTA pH 8.0, 0.5 mM EGTA pH 8.0). Samples were centrifuged at 1100 × *g* for 2 min. Supernatants were collected as nuclear soluble fractions. Pellets were washed once with 1 ml E2 buffer and resuspended in 750 μl E3 buffer (500 mM Tris–HCl pH 6.8, 500 mM NaCl) as chromatin-bound fractions. Chromatin fractions were then sonicated using a Covaris E220 sonicator for cycles of 900 s (15 s on and 15 s off) at 4°C.

To prepare beads bound to antibody, 250 μl Pierce Protein A/G magnetic beads (Thermo Fisher #88803) were added to a 2 ml Eppendorf tube per sample, washed with 1X TBS pH 8.1 (5 ml) and collected with a magnet. Beads were resuspended in 1X TBS (1 ml), with the addition of 30 μg either MORC2 antibody (Bethyl Lab #A300-149A), GFP antibody (Invitrogen #MA5-15256) or Rabbit IgG Isotype Control (Invitrogen #02–6102), and the mixture was incubated at 4°C with end-over-end mixing on a Tube Revolver (Fisher Scientific cat. no. 88861051) for 16–24 h. To immunoprecipitate MORC2, different fractions of cell lysates were incubated with Rabbit-IgG Isotype Control antibody conjugated beads at 4°C for 1 h. Lysate was clarified by centrifugation (3220 × *g*, 30 min, 4°C) and transferred to a new tube. To each lysate, magnetic beads conjugated to anti-MORC2 or anti-GFP antibody were added, and the mixture was incubated at 4°C with end-over-end mixing for 16 h. Beads were washed once with 1 ml of E1 buffer and transferred to a new 2 ml of Eppendorf tube. Beads were further washed once with 1 ml of 300 mM NaCl in 1X TBS and five further times with 1 ml of 1X TBS changing Eppendorf tubes every two washes.

### Phospho-mass spectrometry analysis of MORC2 extracted from HeLa cells

#### Peptide digestion

Protein-bound magnetic beads were resuspended in 100 μl of 100 mM tetraethylammonium bromide (TEAB) with 40 mM CAA and 10 mM TCEP in MS-grade water and reduced and alkylated at 70°C for 15 min in Low Protein Binding Microcentrifuge Tubes (Thermo Fisher Scientific, Waltham, MA, USA). 1 μg trypsin/Lys-C mix was added in 1 μl of 100 mM TEAB and bead suspensions were digested overnight in a shaking incubator at 37°C at 115 RPM. The following day, 1 μg trypsin/LysC mix was added in 1 μl of 100 mM TEAB and the digestion continued at 37°C for 4 h. 10 μg peptides were dried using a speed-vac concentrator and resuspended in 20 μl 100 mM TEAB.

#### Mass spectrometry

Mass spectrometry was performed using an Orbitrap Eclipse mass spectrometer equipped with a FAIMS Pro source connected to an Vanquish Neo nLC chromatography system, all from Thermo Fisher Scientific (Waltham, MA, USA). Peptides were separated using an Aurora Ultimate TS25 column (75 μm × 25 cm, 120 Å) from IonOpticks (Fitzroy, VIC, AUS) at 400 nl/min on a gradient of 3–25% B for 90 min, 25–40% B for 30 min, 40–95% B for 10 min, 95% B over 6 min, using 0.1% formic acid in water for A and 0.1% formic acid in 80% acetonitrile for B. The Orbitrap and FAIMSpro were operated in positive ion mode with a positive ion voltage of 2100 V, an ion transfer tube temperature of 305°C, and a 4.2 L/min carrier gas flow, using standard FAIMS resolution and compensation voltages of -45, –55 and –65 V. Full scan spectra were acquired in profile mode at a resolution of 120 000 (MS1) and 50 000 (MS2), with a scan range of 400–1400 *m/z*, custom maximum fill time (200 ms), custom AGC target (100% MS1, 250% MS2), isolation windows of *m/z* 0.7, intensity threshold of 2.0e4, 2–6 charge state, dynamic exclusion of 60 s and 38% HCD collision energy.

#### Analysis of mass spectrometry data

Raw spectra were analyzed in Proteome Discoverer 2.4 (Thermo Fisher Scientific) to generate protein and peptide identifications using Sequest HT (Thermo Fisher Scientific) and the *Homo sapiens* protein database (UP000005640). For GFP-MORC2 IP-MS experiments, EGFP was included in the search database. The maximum missed cleavage sites for trypsin were limited to 2. Precursor and fragment mass tolerances were 10 ppm and 0.02 Da, respectively. The following post-translational modifications: dynamic phosphorylation (+79.966 Da; S, T, Y), dynamic oxidation (+15.995 Da; M), dynamic acetylation (+42.011 Da; N-terminus), dynamic Met-loss (–131.04 Da; M N-terminus), dynamic Met-loss + acetylation (–89.03 Da; M N-terminus) and static carbamidomethyl (+57.021 Da; C). Peptides identified in each sample were filtered by fixed value PSM validator with a maximum delta *C*n of 0.05.

### Immunofluorescence

HeLa, RPE1, and A549 cells were seeded on poly-L-lysine coated coverslips and fixed in 1 ml of PBS + 4% formaldehyde + 0.25% Triton X-100 at room temperature for 15 min. Following fixation, cells were washed three times with 1 ml of PBS + 0.1% Triton X-100 and blocked in AbDil for 16 h. Following blocking, cells were stained at room temperature for 2 h with 200 μl of α-MORC2 antibody (1:500, A300-149A) diluted in AbDil. Cells were washed with 200 μl of PBS + 0.1% Triton X-100, three times then incubated in 200 μl of Cy5-conjugated secondary antibodies (1:300, Jackson ImmunoResearch Laboratories) in AbDil for 1 h at room temperature. After secondary, cells were stained in 200 μl of 1 μg/ml Hoescht for 15 min at room temp, then washed three times with 200 μl of PBS + 0.1% Triton X-100 before mounting in PPDM (0.5% p-phenylenediamine and 20 mM Tris-Cl, pH 8.8, in 90% glycerol) and sealed with nail polish. Images were taken on the Deltavision Ultra (Cytiva) system using a 60x/1.42NA objective. 8 μm images were taken with *z*-sections of 0.2 μm. All images presented were deconvolved and max projected.

### Fluorescence microscopy

Cells were plated in 12-well glass-bottom plates and treated with 1 μg/ ml doxycycline for 24 h. Medium was replaced with CO_2_-independent medium with 0.1 μg/ ml Hoechst to visualize DNA and incubated on the stage for 30 min before imaging. Cells were maintained at 37°C using a heated stage. Images were taken on a Deltavision Ultra microscope (Cytiva) using a 60×/1.42NA objective. 8 μm images were taken with z sections of 0.2 μm. All images shown are deconvolved and maximally projected (2 μm) using Fiji/ImageJ. For images where the localization of the protein was being assessed, the images were not scaled equivalently (Figure 5B and Supplementary Figure S7F and H). Morphological markers such as DNA were not scaled equivalently.

### RNA extraction and sequencing

Cells were plated in six-well plates and treated with 1 μg/ml doxycycline for 48 h. Cells were washed once with PBS, resuspended 400 μl of TRI Reagent (Invitrogen, AM9738) and stored at −80°C. 120 μl of chloroform was added to the TRI reagent, the mixture was vortexed then separated by centrifugation at 21 000 × *g*, 4°C for 15 min. The aqueous layer containing the nucleic acids was collected. Nucleic acids were precipitated with 300 mM NaCl and 30 μg GlycoBlue (AM9516) by the addition of equal volumes of isopropanol and incubation at −20°C overnight. Precipitated nucleic acids were collected by centrifugation at 21 000 × g, 4°C for 15 min. The supernatant was removed, and the nucleic acid pellet was washed with 70% ethanol and dissolved in RNase-free water. Nucleic acid concentration was quantified using the Qubit RNA Broad Range kit (Q10210).

To generate spike-in mRNAs for absolute RNA quantification in RNA-seq, plasmids containing T7 promoter–Nano or Firefly Luciferase were amplified by PCR. The template for PCR was digested with DpnI then purified using EconoSpin TM All-in-1 Mini Spin Columns (Epoch Life Science, 1920–250) and eluted in RNase free water. 500 ng of the PCR product was used in an *in vitro* transcription reaction using the HiScribe T7 High Yield RNA Synthesis Kit (NEB, E2040S). The DNA template was digested through addition of DNase I after *in vitro* transcription. Free nucleotides were removed using a P30 spin column (Bio Rad, 7326251). Then, the RNA was purified by phenol chloroform extraction and isopropanol precipitation. The RNA was capped using the Vaccinia Capping System (NEB, M2080S), desalted on P30 columns, phenol chloroform extracted and precipitated overnight at −20°C with isopropanol. The capped RNA was polyadenylated using *E. coli* polyA polymerase (NEB, M0276L), desalted on P30 columns, phenol chloroform extracted and precipitated overnight at −20°C with isopropanol. The purified capped and polyadenylated mRNA was resuspended in RNase free water, the Nano and Firefly luciferase was mixed at a ratio of 1:2, aliquoted, flash frozen in liquid nitrogen and stored at −80°C.

For RNA-sequencing, 2 μg of total RNA was supplemented with 5 pg *in vitro* transcribed Nano and Firefly luciferase mRNA. RNA integrity was assessed using Agilent’s Fragment Analyzer prior to library preparation. Sequencing libraries were prepped using the NEBNext Ultra II RNA Library Prep Kit and sequenced using the Element AVITI sequencer with 75 × 75 bp paired end runs.

### Analysis of RNA-sequencing data

A custom genome and annotation containing nano luciferase mRNA, firefly luciferase mRNA, transposable elements (hg38, repeatmasker) and human canonical protein coding mRNAs (hg38, gencode v35) were used for mapping. The custom genome was indexed using STAR (v2.7.1a) (49) with the option –sjdbOverhang 74. As suggested by Tetranscripts (50), reads were aligned to the hg38 reference genome using STAR with options –outFilterMultimapNmax 100 –winAnchorMultimapNmax 200 –outFilterType BySJout –outSAMattributes All –outSAMtype BAM SortedByCoordinate. Mapped .bam files were indexed and reads were quantified using Tetranscripts (v2.2.3) (50) with the following options –sortByPos –format BAM –mode multi –stranded reverse –minread 25. For non-spike normalized data (Supplementary Figure S9E), the DEseq2 output from the TEtranscript package was used, where the default DEseq2 median of ratios method for normalization was performed.

For spike-in mRNA quantification, the mapped reads were quantified using htseq-count (v1.99.2) (51) with the options -r pos –stranded = reverse -f bam -t exon. The sample sizeFactor was calculated by summing counts of nano and firefly luciferase mRNA then normalizing the summed spike mRNA counts to control NLS-GFP replicate 1. These sizeFactors along with raw count outputs from TEtranscripts were used for DEseq2 (52) to calculate adjusted *P*-values and fold changes.

For PCA analysis (Supplementary Figure S9D), the spike normalized DEseq2 data were rlog transformed and PCA plots were generated using plotPCA with default settings from the DEseq2 package. For quantification of MORC2 mRNA expression from RNA sequencing data (Supplementary Figure S9B), the raw counts from TEtranscripts were normalized using DEseq2 median of ratios.

## Results

### MORC2 preferentially associates with double-stranded DNA

**Figure 2.**
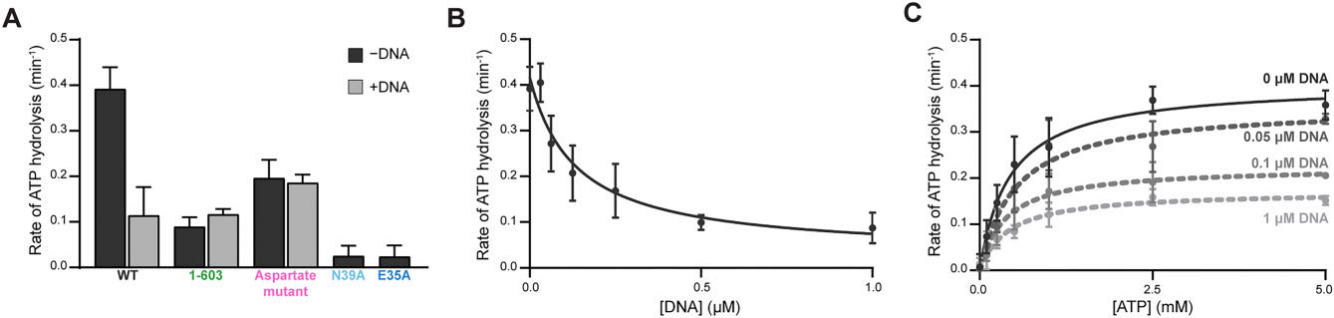
DNA binding by MORC2 reduces ATPase activity. (**A**) Assessment of MORC2 ATPase activity. Dephosphorylated wild-type, 1–603, DNA binding deficient aspartate mutant, and ATP hydrolysis deficient mutant E35A, and ATP binding deficient mutant N39A MORC2 (1 μM) were incubated with 1 mM ATP for 45 min at 37°C either in the presence or absence of a 35 base pair DNA (2 μM). Inorganic phosphate released was quantified by malachite green (‘Materials and methods’ section). Error bars correspond to the standard deviation between three replicate experiments. (**B**) MORC2 ATPase activity is reduced in the presence of DNA. Dephosphorylated MORC2 (1 μM) was incubated with 1 mM ATP for 45 min at 37°C with a titration of a 35 base pair duplex DNA. Inorganic phosphate released was quantified by malachite green (‘Materials and methods’ section). Data were fit to an inhibition curve. Error bars correspond to the standard deviation between three replicate experiments. (**C**) Michaelis–Menten analysis of MORC2 ATPase activity in the presence of DNA. Dephosphorylated MORC2 (1 μM) was incubated with an ATP titration for 45 min at 37°C in the presence of various concentrations of 35mer DNA (0–1 μM). Inorganic phosphate released was quantified by malachite green (‘Materials and methods’ section). Data were fit to a Michaelis–Menten model of enzyme kinetics. Error bars correspond to the standard deviation between three replicate experiments.

**Figure 3.**
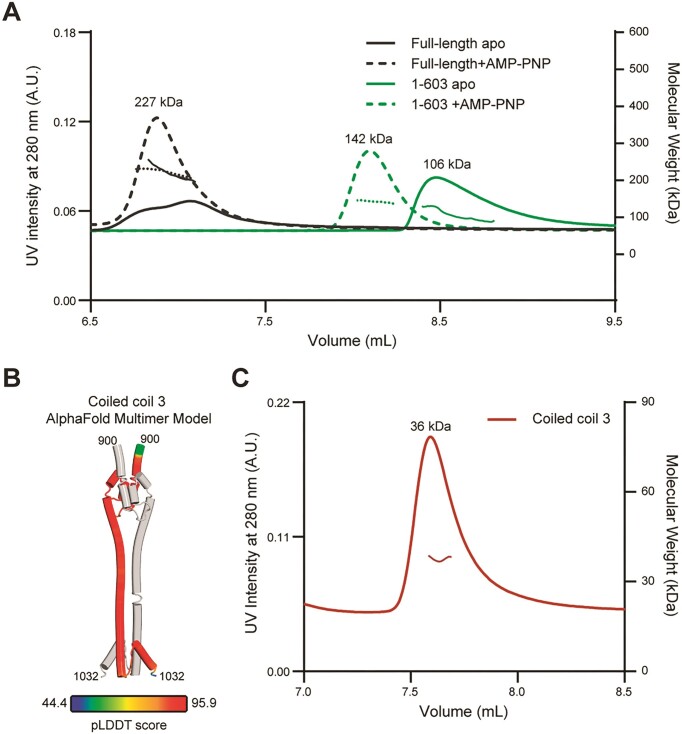
MORC2 homodimerizes via two distinct interfaces. (**A**) Dephosphorylated full-length MORC2 dimerizes in the presence and absence of AMP-PNP. Size-exclusion chromatography coupled to multi-angle light scattering (SEC-MALS) experiments with wild-type (monomer MW = 117 kDa) and 1–603 (monomer MW = 70 kDa) MORC2 in the presence or absence of 1 mM AMP-PNP. 2 mg/ml of MORC2 was applied to a WC-030 column (Wyatt Technology), and elution was monitored by absorption at 280 nm (left *y*-axis). MALS analysis of molecular weight is shown on the right *y*-axis, and average molecular weight calculations are shown across the center of each peak. (**B**) AlphaFold Multimer model of coiled coil 3 domain dimer. Chain A is colored gray and chain B is colored by pLDDT score. (**C**) MORC2 coiled coil 3 dimerizes in solution. SEC-MALS experiment performed with 1.5 mg/ml of purified MORC2 coiled coil 3 (monomer MW = 16 kDa) on a WC-010 column (Wyatt Technology). Elution was monitored by absorption at 280 nm (left *y*-axis). MALS analysis of molecular weight is shown on the right *y*-axis, and the average molecular calculation is shown across the center of the peak.

To characterize the interaction of full-length human MORC2 with nucleic acids, we recombinantly overexpressed human MORC2 using a baculovirus expression system and purified it to homogeneity (Figure 1B and Supplementary Table S1; Supplementary Figure S1A and B; ‘Materials and methods’ section). Heterogeneous phosphorylation of the protein was removed by phosphatase treatment during the purification (‘Materials and methods’ section). The purified protein is dimeric and hydrolyzes ATP (Figures 2 and 3).

MORC family members associate with a wide range of nucleic acid substrates. For example, MORC3 has been shown to bind ssRNA and nucleosomal substrates (19,53). There is also evidence that MORC3 favors specific DNA sequences (8,54) and that *C. elegans* MORC-1 preferentially binds longer DNA substrates (>1000 bp) (15). We thus tested whether MORC2 has preferences for specific nucleic acids or topologies by fluorescence anisotropy (FA) assays using 5′-FAM-labeled nucleic acid substrates or by gel shift assays. We observe that DNA GC content does not substantially affect MORC2 DNA association (Figure 1C). In contrast, MORC2 preferentially associates with longer DNAs (25, 45 and 65 bp substrates tested) (Supplementary Figure S2A and Supplementary Table S2). In a gel shift assay with 500 and 1000 bp DNA substrates, we observed a laddering pattern with increasing concentrations of MORC2, indicating that multiple MORC2 complexes associate with the same piece of DNA (Figure 1D and E and Supplementary Figure S2B).

Next, we tested whether MORC2 prefers dsDNA, ssDNA, ssRNA, cruciform DNA or nucleosomal substrates. Nucleosomes were reconstituted by combining purified *X. laevis* histone octamers and a 149 bp dsDNA with a Widom 601 nucleosome positioning sequence (Supplementary Figure S1C) (55), and cruciform DNA was prepared by annealing four ssDNA oligos (Supplementary Figure S1D) (56). MORC2 most readily associates with double stranded substrates, with a slight preference for the 149 bp Widom 601 dsDNA than the reconstituted nucleosome (149 bp Widom 601 dsDNA: *K*_d, app_ 5 nM or less, nucleosome: *K*_d, app_ 16 ± 2 nM, ) (Figure 1F and Supplementary Table S2). The MORC2 affinity for ssDNA, ssRNA or cruciform DNA is ∼10-fold less than its affinity for dsDNA (ssDNA: *K*_d, app_ 51 ± 9 nM, ssRNA: *K*_d, app_ 49 ± 6 nM, cruciform DNA: *K*_d, app_ 78 ± 12 nM). We note that the binding behavior on the cruciform substrate is complex and was modeled using a Hill equation.

Finally, we assessed MORC2 association with negatively supercoiled, positively supercoiled or relaxed plasmid substrates. We used a competition assay where 1 nM of a 35mer FAM-labeled dsDNA substrate was incubated with 0.15 μM MORC2. Plasmid DNA was titrated and association with plasmid DNA was measured as the loss of fluorescence anisotropy. We observe that MORC2 binds all plasmid substrates with no apparent preference for a topological state (Figure 1G). Together, these results indicate that full-length MORC2 preferentially binds dsDNA in a sequence and topology independent manner.

### A C-terminal region of MORC2 strongly associates with DNA

To identify regions of MORC2 that associate with DNA, we performed mechlorethamine and UV-induced DNA–protein cross-linking with dephosphorylated MORC2 and a 65mer dsDNA oligo (‘Materials and methods’ section). Cross-links were detected by mass spectrometry. Mechlorethamine primarily cross-links purines with amino acids that are 7–8 Å away whereas UV light induces zero-length cross-links between amino acids adjacent to thymidines. We identified regions of MORC2 in both the N- and C-terminus that form multiple cross-links to DNA with both cross-linking approaches (Figure 1A; Supplementary Table S3 and Supplementary Figure S3). Notably, some N-terminal cross-links surround a previously identified region in coiled coil 1 (residues 326–333) that has been shown to associate with DNA (18). To test the relative DNA binding contributions of the N- and C- terminus, we purified an N-terminal fragment of MORC2 (residues 1–603). The N-terminal MORC2 fragment binds DNA weakly (*K*_d, app_ 446 ± 119 nM) as previously reported (Figure 1H) (18).

Four of the C-terminal cross-linking sites abut a positively charged region bearing 12 highly conserved lysine and arginine residues (K704, R707, K713, K716, K721, K722, R754, R755, K756, R758, K760, R761), suggesting that these residues could mediate DNA binding (Figure 1A and Supplementary Figure S4). To test this idea, we mutated these residues to either aspartate or alanine and purified the resulting full-length mutants. Both the aspartate and alanine mutant bind DNA with greatly reduced affinities compared to the wild-type protein (aspartate mutant: *K*_d, app_ 380 ± 120 nM, alanine mutant: *K*_d, app_ 350 ± 60 nM) (Figure 1H and Supplementary Figure S2C). We next mutated subsets of the positively charged residues to aspartate (N-terminal subset [subset N]: K704, R707, K713, K716, K721, K722 and C-terminal subset [subset C)] R754, R755, K756, R758, K760, R761). Partial removal of the positively charged residues results in an intermediate reduction in MORC2 DNA association (subset N: *K*_d, app_ 90 ± 20 nM, subset C: *K*_d, app_ 60 ± 20 nM) (Supplementary Figure S2C). We confirmed that all mutants appear to be well-folded and can hydrolyze ATP (Supplementary Figures S1B and S2D). Together, these results suggest that a region corresponding to MORC2 704–761 is required for strong DNA association.

### MORC2 ATP hydrolysis activity is reduced in the presence of DNA

The rate of ATP hydrolysis by DNA transacting ATPases can be positively or negatively influenced by their association with DNA (19–20,57–60). We thus tested whether DNA affects MORC2 ATP hydrolysis activity. To do this, we utilized a malachite green endpoint assay that detects the release of inorganic phosphate. The MORC2 1–603 construct hydrolyzes ATP with a rate of 0.09 ± 0.02 min^−1^, which is in agreement with prior work (Figure 2A) (18). Full-length MORC2, in the absence of DNA, hydrolyzes ATP four times faster than MORC2 1–603 (0.39 μM ± 0.05 min^−1^). Mutations which should either alter ATP binding (N39A) or hydrolysis (E35A) result in almost no detectable ATPase activity (Figure 2A) (18,61). We next included our 35mer dsDNA oligo at a 4:1 molar ratio DNA:MORC2 dimer in the assay and observed an almost 4-fold reduction in MORC2 ATPase rate (0.11 ± 0.06 min^−1^). DNA inhibits MORC2’s ATPase rate in a dose-dependent manner (DNA IC_50_ 0.14 ± 0.05 μM, [MORC2 dimer] = 0.5 μM) (Figure 2B). In contrast, DNA does not appear to influence the rate of ATP hydrolysis by MORC2 1–603 (0.12 ± 0.01 min^−1^) or the DNA binding deficient aspartate MORC2 mutant (without DNA 0.20 ± 0.04 min^−1^, with DNA 0.19 ± 0.02 min^−1^) (Figure 2A).

We next tested how DNA affects the kinetics of ATP hydrolysis by MORC2 by measuring *K*_m, app ATP_ and *V*_max_ of the enzyme with varying concentrations of DNA. As DNA concentration increases, *K*_m, app ATP_ remains constant whereas *V*_max_ decreases (Figure 2C and Supplementary Table S4). Our *K*_m, app ATP_ for full-length MORC2 corresponds to the previously reported *K*_m, app ATP_ for MORC2 1–603 (18). Together, these results suggest that DNA non-competitively inhibits MORC2 ATPase activity, and DNA binding may induce a conformational change in MORC2 that results in reduced ATP hydrolysis.

Finally, since MORC2 ATP hydrolysis is reduced in the presence of DNA, we tested whether ATP or ATP analogs affect DNA association with MORC2. All tested nucleotides result in a ∼5–50-fold reduction in DNA binding affinity (Supplementary Figure S2E and Supplementary Table S2). Intriguingly, ATPγS leads to the largest reduction in DNA binding affinity. It has been shown that the MORC3 GHKL dimer interface is most strongly stabilized by ATPγS (62). One possible explanation for these observations is that dimerization of the GHKL domain in the presence of ATP or ATP analogs limits MORC2 association with DNA.

### MORC2 contains N- and C- terminal dimerization interfaces

In addition to their N-terminal GHL domain, GHL-type ATPases contain a second, C-terminal dimerization interface (63). To determine the oligomeric state of MORC2 1–603 and full-length MORC2, we utilized SEC-MALS. We observe that the MORC2 1–603 only dimerizes in the presence of ATP or ATP analogs, as previously observed (18). In contrast, full-length MORC2 forms a dimer in both the presence and absence of the ATP analog AMP-PNP. These results suggest the presence of a second, C-terminal dimerization interface (Figure 3A). An AlphaFold multimer prediction of the full-length, dimer structure indicates that the C-terminal coiled coil 3 domain could form an additional dimer interface (pLDDT > 90) (residues 900–1032) (Figure 3B and Supplementary Figure S5) (21). Supporting this model, we observe that the isolated MORC2 C-terminus (residues 900–1032) forms a dimer in solution (Figure 3C). These data indicate that MORC2 contains N- and C- terminal dimerization interfaces like other GHL-type ATPases.

### MORC2 captures circular DNA substrates

**Figure 4.**
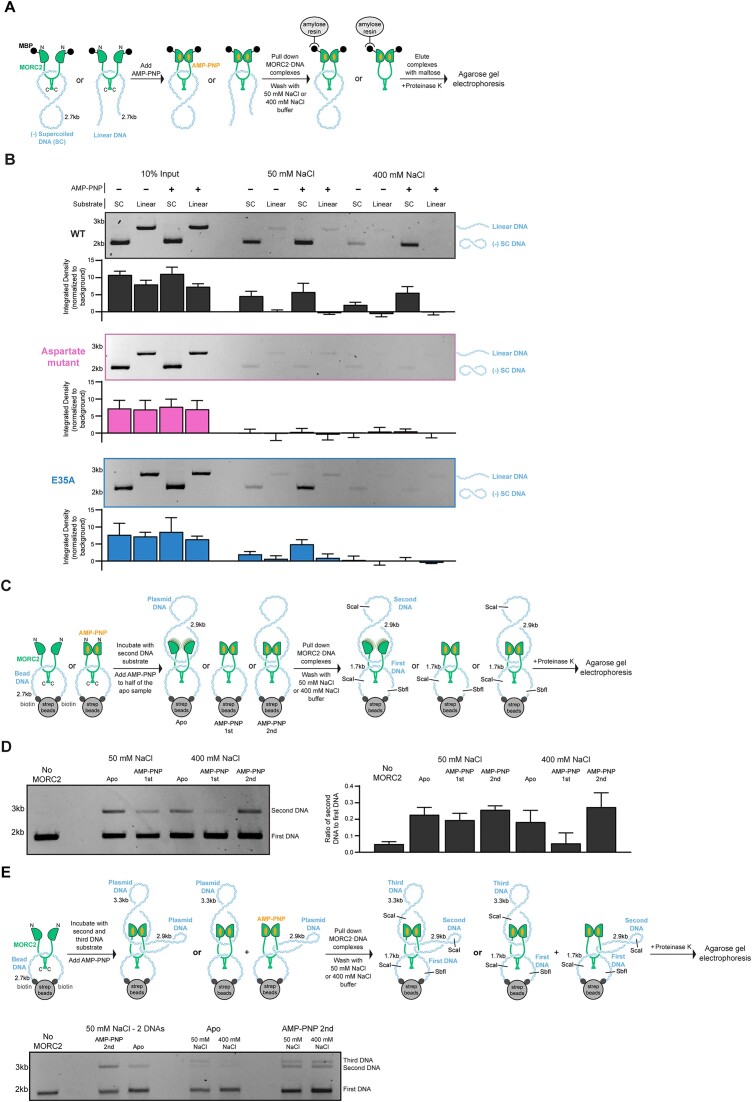
MORC2 can capture circular DNA substrates. (**A**) Schematic of assay to assess MORC2 capture of linear or circular DNA substrates. (**B**) Dephosphorylated, N-terminally-MBP-tagged wild-type, aspartate mutant and E35A MORC2 (600 nM) were incubated with supercoiled or linear pUC19 (100 nM). Samples were then added to amylose resin and washed in either low salt (50 mM NaCl) or high salt (400 mM NaCl) containing buffers before eluting the samples from the beads with maltose. Eluted samples were treated with proteinase K. DNA was resolved on a 1% (w/v) TAE agarose gel. Quantification of the band intensity from input and retained DNA bands normalized to the background are shown below each gel. Error bars correspond to the standard deviation between three replicate experiments. (**C**) Schematic of assay to assess MORC2 association with two circular DNA substrates. (**D**) A biotin-tagged DNA was conjugated to streptavidin magnetic beads to create a pseudo circular substrate. Dephosphorylated MORC2 (600 nM) was incubated with 20 μl of the beads in the presence or absence of 1 mM AMP-PNP. Supercoiled pBlueScript plasmid DNA (200 nM) was added, and 1 mM AMP-PNP was added or omitted before washing the beads with either low salt (50 mM NaCl) or high salt (400 mM NaCl) containing buffer. Samples were resuspended in 1X CutSmart buffer (New England Biolabs). DNA was released from the beads by digestion with ScaI and SbfI at 37°C for 1 h before proteinase K treatment. DNA was resolved on a 1% (w/v) TAE agarose gel. The intensity of the first DNA and second DNA substrate bands were quantified, normalized to the background and are presented as a ratio of second DNA:first DNA band intensity. Error bars correspond to the standard deviation between three replicate experiments. (**E**) Schematic of assay to assess MORC2 association with three circular DNA substrates. A biotin-tagged DNA was conjugated to streptavidin magnetic beads to create a pseudo-circular substrate. Dephosphorylated MORC2 (600 nM) was incubated with 20 μl of the beads. Supercoiled pBlueScript plasmid DNA (100 nM) and pBlueScript-601 plasmid DNA (100 nM) were added and 1 mM AMP-PNP was added or omitted before washing the beads with either low salt (50 mM NaCl) or high salt (400 mM NaCl) containing buffer. Samples were resuspended in 1X CutSmart buffer (New England Biolabs). DNA was released from the beads by digestion with ScaI and SbfI at 37°C for 1 h before proteinase K treatment. DNA was resolved on a 1% (w/v) TAE agarose gel.

Dimerization of GHL-type ATPases at their N- and C- termini can result in the formation of a central substrate binding lumen (63). To test whether MORC2 engages DNA via a central lumen, we utilized MBP-tagged MORC2 in a pull-down assay with plasmid derived circular and linear DNA substrates (Figure 4A). If DNA is engaged inside a central binding lumen, it is expected that circular DNA will not be able to leave the protein when washed with high ionic strength solutions whereas linear DNA will be lost under these conditions because it is not topologically entrapped by the protein. MBP-MORC2 was incubated with linear or circular DNA and then added to amylose beads in the absence or presence of the ATP analog AMP-PNP. The amylose beads were washed extensively with either a low or high ionic strength containing solution (50 or 400 mM NaCl, respectively). MBP-MORC2 was eluted from the beads with maltose, digested with proteinase K, and the retained DNA was collected and separated by agarose gel electrophoresis. Circular DNA substrates are preferentially captured relative to linear substrates (∼30 fold) after a low ionic strength wash (Figure 4B and Supplementary Figure S6A). The addition of AMP-PNP does not substantially affect DNA retention under these conditions. In contrast, after a high ionic strength wash, retention of circular DNA substrates is enhanced with the inclusion of AMP-PNP, indicating that robust DNA retention requires the engagement of both dimerization interfaces. We also tested the ability of the aspartate DNA binding mutant and the ATP hydrolysis deficient E35A mutant to retain DNA (Figure 4B and Supplementary Figure S6A). The aspartate mutant captured almost no DNA under any condition. The E35A mutant retained circular substrates in the presence of AMP-PNP after low ionic strength washes. This effect was lost after high ionic strength washes, likely because the E35A GHKL domain dimerizes less stably (61). These results indicate that DNA binds within a central MORC2 lumen and that the DNA binding surface between residues 704 and 761 is required for this interaction.

### MORC2 can bridge two DNA substrates

MORC family proteins are proposed to compact DNA by engaging multiple strands of DNA simultaneously (15). We assessed if MORC2 can bind multiple DNA strands using a dual DNA pulldown assay (Figure 4C). A DNA substrate containing ScaI and Sbf1 restriction sites was biotinylated on both ends and applied to streptavidin-coated magnetic beads to form a pseudo-circular DNA substrate. MORC2 was incubated with the pseudo-circular DNA substrate, and AMP-PNP was added or omitted to either stabilize the N-terminal dimerization interface or allow for its transient opening. Next, a second, circular plasmid DNA substrate with a single ScaI restriction site was added, and AMP-PNP was added or omitted to stabilize the N-terminal dimerization interface around the DNA substrates. All samples were washed with either low or high ionic strength containing solutions. DNA was released from the beads by ScaI and Sbf1 restriction enzyme digest resulting in 1.7 and 2.9 kb DNA fragments corresponding to the biotinylated DNA and plasmid DNA species, respectively. MORC2 was digested with proteinase K, and the resulting DNA was collected and separated by agarose gel electrophoresis.

Control experiments without MORC2 show no retention of the second plasmid substrate. Under low ionic strength conditions, MORC2 associates with both DNA substrates efficiently in an AMP-PNP independent manner (Figure 4D and Supplementary Figure S6B). A high ionic strength wash, however, shows that MORC2 cannot effectively associate with the second plasmid DNA substrate when AMP-PNP is added before the second plasmid DNA substrate (Figure 4D ‘AMP-PNP 1^st^’ and Supplementary Figure S6B). When AMP-PNP is added after the second plasmid DNA substrate, we observed enhanced retention of the second plasmid DNA (Figure 4D ‘AMP-PNP 2^nd^’ and Supplementary Figure S6B). These results suggest that stable, second strand capture requires the opening of the N-terminal GHL dimerization interface.

To test whether MORC2 can capture multiple DNA substrates, we repeated the experiment with the addition of a larger, third plasmid DNA substrate with a single ScaI restriction site. After both low and high ionic strength washes, we observe retention of both the second and third DNA substrates (Figure 4E and Supplementary Figure S6C). Although this experiment cannot distinguish between a single MORC2 dimer capturing three DNA substrates or a population of different MORC2 dimers capturing two DNA substrates each, this experiment indicates that MORC2 likely could capture two or more circular DNA substrates simultaneously.

### Phosphorylation of MORC2 reduces its association with DNA

Phosphorylation site analysis by mass spectrometry indicates that our heterologously overexpressed and purified MORC2 is heterogeneously phosphorylated during its expression in insect cells, particularly in the C-terminal region, and thus we removed these phosphorylations to obtain homogeneous protein for our biochemical studies (‘Materials and methods’ section and Supplementary Figure S7A). To define the phosphorylation state of MORC2 in human cells, we immunoprecipitated MORC2 from HeLa cells and subjected it to mass spectrometry analysis. We observe that MORC2 is heterogeneously phosphorylated with 8 out of 17 phosphorylation sites being shared between insect cell overexpression and HeLa cells, respectively (Supplementary Figure S7A and B and Supplementary Table S5).

**Figure 5.**
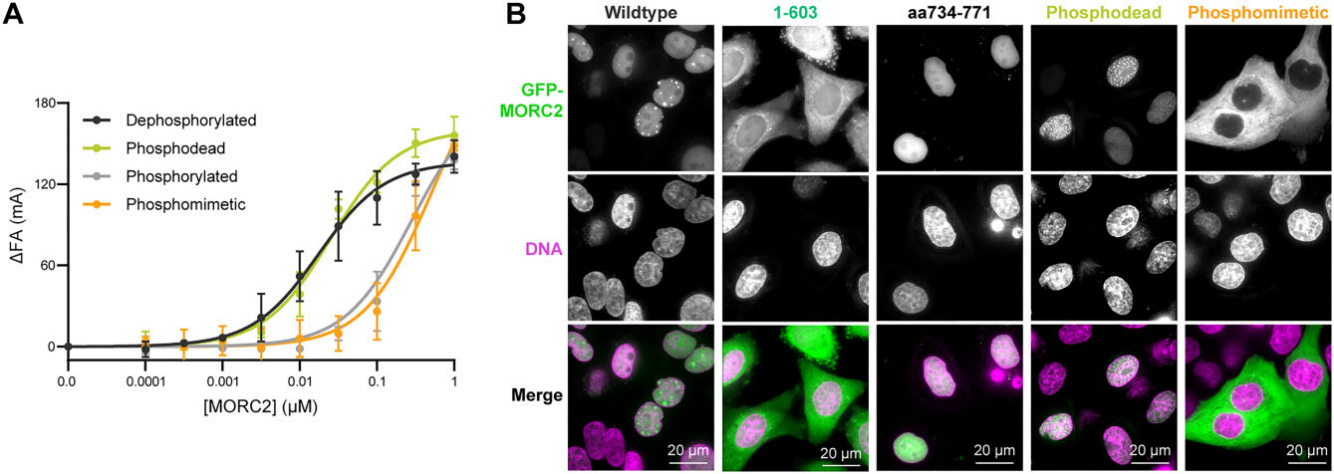
MORC2 phosphorylation influences DNA binding and nuclear localization. (**A**) Phosphorylation reduces MORC2 affinity for DNA. Dephosphorylated, phosphorylated, phosphodead and phosphomimetic MORC2 were assessed for DNA binding using fluorescence anisotropy. MORC2 was titrated with FAM-labeled 35 base pair duplex DNA (1 nM) (‘Materials and methods’ section). Binding curves were fit using a quadratic binding equation. Error bars correspond to the standard deviation between three replicate experiments. (**B**) Effect of phosphorylation mutations on MORC2 localization in interphase HeLa cells. Representative confocal microscopy images of interphase HeLa cells overexpressing EGFP-wild-type MORC2, EGFP-MORC2 1–603, EGFP- MORC2 734–771, EGFP- phosphodead MORC2 and EGFP- phosphomimetic MORC2.

We tested how phosphorylation affects MORC2 association with DNA using FA with a 35mer FAM-labeled dsDNA substrate. *In vitro* dephosphorylated MORC2 binds DNA with ∼10 fold greater affinity than MORC2 preparations where phosphorylations installed during insect cell expression remain intact (phosphorylated: *K*_d, app_ 285 ± 83 nM, dephosphorylated: *K*_d, app_ 17 ± 4 nM) (Figure 5A).

To understand how comprehensive phosphorylation of the MORC2 C-terminal region affects DNA binding, we selected 17 MORC2 C-terminal region phosphorylation sites that were identified here in HeLa cells or were previously identified in other human cells lines (S615, T650, S696, S703, S705, S711, T717, T723, S725, S730, T733, S735, S739, S743, S773, S777, S779) (Figure 1A and Supplementary Figure S4) (4,64). Interestingly, three phosphorylation sites (S725, S730 and S773) were identified as residues under positive selection during primate evolution (31). We mutated the seventeen identified serine and threonine phosphorylation sites to aspartate and glutamate, respectively, to mimic the expected charge in a fully phosphorylated state (phosphomimetic) or to alanine to prevent phosphorylation (phosphodead). Both mutants appear to be well-folded and hydrolyze ATP (Supplementary Figures S1B and S2D). The phosphomimetic mutant has a greatly reduced affinity for DNA (*K*_d, app_ 512 ± 147 nM) whereas alanine substitutions marginally affect DNA association (*K*_d, app_ 25 ± 3 nM) (Figure 5A). Together, these results suggest that extensive phosphorylation of MORC2 in the region between residues 603 and 790 likely impairs DNA association.

### MORC2 C-terminus is required for nuclear localization

We next explored how our biochemically identified DNA binding sites and putative phosphorylation sites affect MORC2 localization in cells. Previous reports have shown that MORC2 primarily localizes to the nucleus (65,66), and we see similar localization patterns in human HeLa, RPE1 and A549 cells (Supplementary Figure S7C and D). MORC2 contains two putative nuclear localization sequences. One lies in the MORC2 C-terminus, corresponding to MORC2 residues 755–762 (Supplementary Figure S7E) (67), and the second is found in coiled-coil 1 (residues 344–364) (Supplementary Figure S7E). To do define which NLS is most critical for MORC2 localization, we introduced EGFP tagged MORC2 ectopically into HeLa cells using the piggyBac transposase system (‘Materials and methods’ section). EGFP-MORC2 expression was induced with doxycycline for 48 h. EGFP attached to MORC2 residues 734–771 localizes to the nucleus (Figure 5B), whereas EGFP-MORC2 1–603 is mostly found in the cytoplasm (Figure 5B), suggesting that the C-terminal NLS primarily drives MORC2 nuclear localization. We note that in our aspartate DNA binding mutant, we directly substituted positively charged residues in the C-terminal NLS, and observe that this construct localizes to the cytoplasm, further supporting the identified C-terminal NLS (Supplementary Figure S7F).

We next investigated how MORC2 phosphorylation may affect MORC2 cellular localization using our phosphodead and phosphomimetic MORC2 mutants. We note that overexpressed MORC2 is phosphorylated to a similar extent as the endogenous protein and that overexpressed MORC2 forms distinct puncta in the nucleus as observed for MORC2 and other MORC family members (3,66,72, Human Protein Atlas) (Figure 5A and Supplementary Figure S7F and G and Supplementary Table S6). Wild-type MORC2 and phosphodead MORC2 localized to the nucleus (Figure 5B). In contrast, phosphomimetic MORC2 is found in the cytoplasm (Figure 5B). These results suggest that phosphorylation of the MORC2 C-terminus may regulate MORC2 localization.

### The C-terminal DNA binding region is required for MORC2 gene silencing in cells

**Figure 6.**
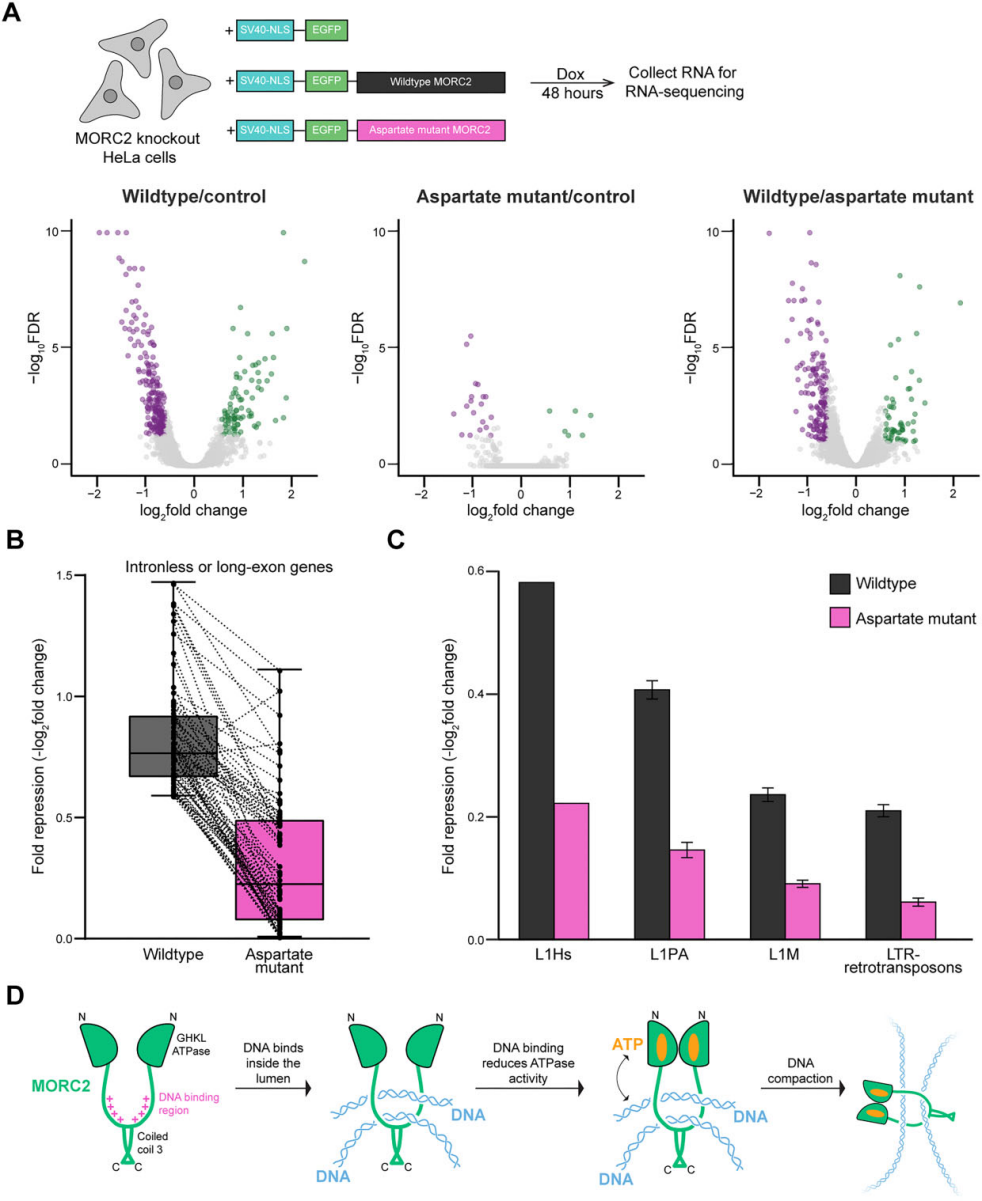
DNA binding regulates MORC2 gene silencing activity in cells. (**A**) Schematic of RNAseq experiment. MORC2 constructs were added to HeLa cells lacking MORC2. MORC2 expression was induced for 48 h with doxycycline after which RNAs were extracted and sequenced. Volcano plots of RNAseq reads for overexpression of wild-type MORC2 versus control, overexpression of aspartate mutant MORC2 versus control, and wild-type MORC2 versus aspartate mutant MORC2 with spike normalization (‘Materials and methods’ section). Significant upregulated genes are shown in green, and significant downregulated genes are shown in purple from three biological replicates. Significant genes are classified as those that meet the fold change > 1.5 and FDR > 0.05 cutoffs. (**B**) Fold repression (-log_2_fold change) analysis of intronless and long-exon containing genes exhibiting a significant degree of repression after overexpression of wild-type MORC2 (*n* = 75) in comparison to their fold repression after overexpression of aspartate mutant MORC2. Error bars correspond to the minimum and maximum values and the central bar represents the median value. Dotted lines connect genes between the two conditions. (**C**) Average fold repression (-log_2_fold change) of retrotransposon subfamilies as measured by RNAseq in cells with overexpression of wild-type or aspartate mutant MORC2 versus control cells using the TEtranscripts tool. Error bars represent the standard error between the elements in each subfamily (L1Hs *n* = 1, L1PA *n* = 30, L1M *n* = 86 and LTR-retrotransposons *n* = 347). (**D**) Model of how MORC2 engages DNA to promote compaction. MORC2 contains a DNA binding region between the GHKL domain and coiled coil 3 domain dimerization interfaces. DNA binding inside the lumen of the dimer communicates to the ATPase domain, to favor an ATP-bound homodimer conformation that is incompatible with ATP hydrolysis. MORC2 association with multiple DNA segments may allow MORC2 to bridge distal regions of DNA to contribute to compaction.

We next tested how the MORC2 DNA binding region affects MORC2’s silencing function in cells. We appended an artificial SV40 NLS sequence (35) to EGFP, EGFP-wild-type MORC2 and the EGFP-MORC2 DNA binding deficient aspartate mutant to ensure all MORC2 proteins were localized to the nucleus (Supplementary Figure S7H and I and Supplementary Table S7), and ectopically introduced these constructs into a MORC2 knockout HeLa cell line (‘Materials and methods’ section and Supplementary Figure S8). Doxycycline was added to induce the overexpression of EGFP or EGFP-MORC2 for 48 h, and total RNA was collected for spike-in normalized RNA sequencing (‘Materials and methods’ section; Figure 6A and Supplementary Figure S9). The exogenous EGFP-MORC2 protein was expressed at levels higher than endogenous MORC2 (Supplementary Figure S9B). We confirmed that expression levels of MORC2 were similar between the wild-type and aspartate mutant samples (Supplementary Figure S9C). Analysis of human protein-coding genes shows that overexpression of wild-type EGFP-MORC2 leads to significant upregulation of 69 genes and significant downregulation of 197 genes in comparison to control cells expressing EGFP (Figure 6A and Supplementary Table S8 and Supplementary Figure S9E and F). Almost 40% of the genes downregulated by wild-type MORC2 are intronless or contain exons longer than 1 kb, of which 26 are zinc finger (ZNF) genes, consistent with prior studies (Supplementary Figure S9F and G) (5). In contrast, overexpression of EGFP-tagged aspartate mutant MORC2 leads to significant upregulation of 4 genes and significant downregulation of 13 genes in comparison to control cells expressing EGFP (Figure 6A). We compared the fold repression of intronless or long-exon containing genes that are significantly repressed by wild-type MORC2 with their fold repression after expression of the aspartate mutant, and the aspartate mutant exhibits weaker repression for almost all genes analyzed (Figure 6B).

We next assessed the role of MORC2 in silencing retrotransposons using the TEtranscripts tool, which improves mapping of transposable elements (50). Expression of wild-type MORC2 represses evolutionarily young retrotransposons as previously observed (28,32) whereas expression of the DNA binding deficient aspartate mutant weakly represses expression of retrotransposons (Figure 6C). The most highly repressed retrotransposons are the youngest and second youngest LINE-1 classes in the human genome, L1Hs and L1PA, respectively. Wild-type MORC2 weakly represses expression of older LINE-1s from the L1M class and LTR-retrotransposons. Taken together, these results indicate that the MORC2 C-terminal DNA binding region is required for silencing known MORC2 targets.

## Discussion

Here we have identified a MORC2 DNA binding region (residues 704–761) that lies in between its GHKL domain and C-terminal domain dimerization interfaces. This region is required for the capture of circular DNA substrates by MORC2, indicating that MORC2 can topologically entrap DNA. Notably, DNA binding reduces MORC2 ATP hydrolysis activity. We also observe that MORC2 is phosphorylated, and this phosphorylation could regulate MORC2 cellular localization and association with DNA. Finally, we show that the identified DNA binding region is required for MORC2 gene silencing in HeLa cells.

Our data integrated with previous results provide a model for how MORC2 could promote chromatin compaction and gene silencing (Figure 6D). In this model, transient opening of the MORC2 GHKL domain would allow for DNA substrates to enter the MORC2 lumen and associate with the MORC2 C-terminal DNA binding region. DNA binding is communicated to the GHKL domain to presumably favor an ATP-bound GHKL homodimer conformation that is incompatible with ATP hydrolysis. This would serve to topologically clamp MORC2 around DNA, by prolonging the ATP-bound homodimerized state of the GHKL domain. The mechanism for coupling between the ATPase and C-terminal DNA binding region is presently unclear. Association of MORC2 with multiple DNA segments, as we observe here, could lead to compaction of the DNA by bringing distal DNA segments together and thereby promote gene silencing.

The concept that ATPase rate and MORC2 silencing activity are coupled is borne out in neuropathic disease associated MORC2 mutants that alter GHKL dimerization efficiency and ATP hydrolysis rates (18). Specifically, MORC2 S87L, a mutation associated with Charcot–Marie–Tooth disease (CMT) stabilizes GHKL dimerization and has negligible ATPase activity and high levels of silencing in cells. Similarly, a MORC2 R252W substitution also associated with CMT has reduced ATPase activity and elevated levels of silencing in cells (18). MORC2 T424R is correlated with spinal muscular atrophy, reduces GHKL dimerization, has high ATPase levels, and reduced levels of silencing compared to the wild-type protein (18,68,69)^69^. Thus, ATP hydrolysis rate appears to be inversely associated with MORC2 silencing activity in cells, and our results indicate that DNA binding leads to reduced ATPase activity. In sum, MORC2 ATPase activity appears to be carefully tuned to support appropriate MORC2 function in cells.

These results support a general mechanism for how MORC family proteins engage with DNA to promote gene silencing. All MORC family proteins contain an N-terminal GHKL domain and a predicted C-terminal dimerization domain (1,16). Dimerization of MORC proteins around DNA at both interfaces could result in topological entrapment of chromatin substrates. Indeed, *C. elegans* MORC-1 and human MORC2 appear to topologically engage with DNA (15). It is not yet known if MORC3 and MORC4 can topologically entrap DNA, and if the DNA binding surface we identified on MORC2 extends to the other MORC family members. From our work, it is also unclear how the previously identified MORC2 coiled coil 1 DNA interaction is used to promote gene silencing (18). This domain is absent in MORC3 and MORC4. It is possible that the coiled coil 1 interface is required for localization but not for topological entrapment of DNA substrates.

MORC family proteins are localized to unique genomic positions. Our results suggest that MORC2 does not bind DNA in a sequence-specific manner. Rather than recognizing a specific motif, MORC2 can be directed to retroelements and intronless genes, in part, through its association with the Human Silencing Hub (HUSH) complex (5). In addition to protein complexes, histone post-translational modifications can be used to localize MORC proteins to specific genomic regions. Histone H3 trimethylation of lysine 4 is associated with MORC3 and MORC4 localization, driven through an interaction with the modified histone tail and the MORC CW domain (8). MORC1 and MORC2 do not engage with histone proteins in a specific manner, indicating their recruitment to specific genomic regions is likely independent of chromatin modification state (33). Future work with full-length MORC proteins will uncover whether all members use a similar mechanism as MORC2 to associate with chromatin and define how MORC family proteins are localized to specific genomic locations to induce silencing. We note that a preprint with highly complementary results was posted while this manuscript was under review (70).

## Supplementary Material

gkae1273_Supplemental_File

## Data Availability

Raw data for RNA sequencing is available under Gene Expression Omnibus [accession code GSE262311]. The mass spectrometry proteomics data have been deposited to the ProteomeXchange Consortium via the PRIDE partner repository [dataset identifier PXD050648]. All reagents generated in this study are available upon reasonable request and will be fulfilled by the corresponding author (SMV).

## References

[1] Moissiard G. , Cokus S.J., Cary J., Feng S., Billi A.C., Stroud H., Husmann D., Zhan Y., Lajoie B.R., McCord R.P. et al. MORC family ATPases required for heterochromatin condensation and gene silencing. Science. 2012; 336:1448–1451. 22555433 10.1126/science.1221472 PMC3376212

[2] Iyer L.M. , Abhiman S., Aravind L. MutL homologs in restriction-modification systems and the origin of eukaryotic MORC ATPases. Biol. Direct. 2008; 3:8. 18346280 10.1186/1745-6150-3-8 PMC2292703

[3] Mimura Y. , Takahashi K., Kawata K., Akazawa T., Inoue N. Two-step colocalization of MORC3 with PML nuclear bodies. J. Cell Sci. 2010; 123:2014–2024. 20501696 10.1242/jcs.063586

[4] Li D.-Q. , Nair S.S., Ohshiro K., Kumar A., Nair V.S., Pakala S.B., Reddy S.D.N., Gajula R.P., Eswaran J., Aravind L. et al. MORC2 signaling integrates phosphorylation-dependent, ATPase-coupled chromatin remodeling during the DNA damage response. Cell Rep. 2012; 2:1657–1669. 23260667 10.1016/j.celrep.2012.11.018 PMC3554793

[5] Tchasovnikarova I.A. , Timms R.T., Douse C.H., Roberts R.C., Dougan G., Kingston R.E., Modis Y., Lehner P.J. Hyperactivation of HUSH complex function by Charcot–Marie–Tooth disease mutation in MORC2. Nat. Genet. 2017; 49:1035–1044. 28581500 10.1038/ng.3878 PMC5493197

[6] Zhang L. , Li D.-Q. MORC2 regulates DNA damage response through a PARP1-dependent pathway. Nucleic Acids Res. 2019; 47:8502–8520. 31616951 10.1093/nar/gkz545 PMC6895267

[7] Liu H.-Y. , Liu Y.-Y., Yang F., Zhang L., Zhang F.-L., Hu X., Shao Z.-M., Li D.-Q. Acetylation of MORC2 by NAT10 regulates cell-cycle checkpoint control and resistance to DNA-damaging chemotherapy and radiotherapy in breast cancer. Nucleic Acids Res. 2020; 48:3638–3656. 32112098 10.1093/nar/gkaa130 PMC7144926

[8] Groh S. , Milton A.V., Marinelli L.K., Sickinger C.V., Russo A., Bollig H., de Almeida G.P., Schmidt A., Forné I., Imhof A. et al. Morc3 silences endogenous retroviruses by enabling Daxx-mediated histone H3.3 incorporation. Nat. Commun. 2021; 12:5996. 34650047 10.1038/s41467-021-26288-7 PMC8516933

[9] Liu J. , Hanne J., Britton B.M., Bennett J., Kim D., Lee J.-B., Fishel R. Cascading MutS and MutL sliding clamps control DNA diffusion to activate mismatch repair. Nature. 2016; 539:583–587. 27851738 10.1038/nature20562 PMC5845140

[10] Verba K.A. , Wang R.Y.-R., Arakawa A., Liu Y., Shirouzu M., Yokoyama S., Agard D.A Atomic structure of Hsp90-Cdc37-Cdk4 reveals that Hsp90 traps and stabilizes an unfolded kinase. Science. 2016; 352:1542–1547. 27339980 10.1126/science.aaf5023 PMC5373496

[11] Laponogov I. , Pan X.-S., Veselkov D.A., Skamrova G.B., Umrekar T.R., Fisher L.M., Sanderson M.R. Trapping of the transport-segment DNA by the ATPase domains of a type II topoisomerase. Nat. Commun. 2018; 9:2579. 29968711 10.1038/s41467-018-05005-x PMC6030046

[12] Ortega J. , Lee G.S., Gu L., Yang W., Li G.-M. Mispair-bound human MutS–MutL complex triggers DNA incisions and activates mismatch repair. Cell Res. 2021; 31:542–553. 33510387 10.1038/s41422-021-00468-y PMC8089094

[13] Lee K. , Thwin A.C., Nadel C.M., Tse E., Gates S.N., Gestwicki J.E., Southworth D.R. The structure of an Hsp90-immunophilin complex reveals cochaperone recognition of the client maturation state. Mol. Cell. 2021; 81:3496–3508. 34380015 10.1016/j.molcel.2021.07.023 PMC8418782

[14] Xie H.-Y. , Zhang T.-M., Hu S.-Y., Shao Z.-M., Li D.-Q. Dimerization of MORC2 through its C-terminal coiled-coil domain enhances chromatin dynamics and promotes DNA repair. Cell Commun. Signal. 2019; 17:160. 31796101 10.1186/s12964-019-0477-5 PMC6892150

[15] Kim H. , Yen L., Wongpalee S.P., Kirshner J.A., Mehta N., Xue Y., Johnston J.B., Burlingame A.L., Kim J.K., Loparo J.J. et al. The gene-silencing protein MORC-1 topologically entraps DNA and forms multimeric assemblies to cause DNA compaction. Mol. Cell. 2019; 75:700–710. 31442422 10.1016/j.molcel.2019.07.032 PMC6814019

[16] Chen K. , Birkinshaw R.W., Gurzau A.D., Wanigasuriya I., Wang R., Iminitoff M., Sandow J.J., Young S.N., Hennessy P.J., Willson T.A. et al. Crystal structure of the hinge domain of Smchd1 reveals its dimerization mode and nucleic acid–binding residues. Sci. Signal. 2020; 13:eaaz5599. 32546545 10.1126/scisignal.aaz5599

[17] Uhlén M. , Fagerberg L., Hallström B.M., Lindskog C., Oksvold P., Mardinoglu A., Sivertsson Å., Kampf C., Sjöstedt E., Asplund A. et al. Tissue-based map of the human proteome. Science. 2015; 347:1260419. 25613900 10.1126/science.1260419

[18] Douse C.H. , Bloor S., Liu Y., Shamin M., Tchasovnikarova I.A., Timms R.T., Lehner P.J., Modis Y. Neuropathic MORC2 mutations perturb GHKL ATPase dimerization dynamics and epigenetic silencing by multiple structural mechanisms. Nat. Commun. 2018; 9:651. 29440755 10.1038/s41467-018-03045-x PMC5811534

[19] Zhang Y. , Klein B.J., Cox K.L., Bertulat B., Tencer A.H., Holden M.R., Wright G.M., Black J., Cardoso M.C., Poirier M.G. et al. Mechanism for autoinhibition and activation of the MORC3 ATPase. Proc. Natl Acad. Sci. U.S.A. 2019; 116:6111–6119. 30850548 10.1073/pnas.1819524116 PMC6442546

[20] Tencer A.H. , Cox K.L., Wright G.M., Zhang Y., Petell C.J., Klein B.J., Strahl B.D., Black J.C., Poirier M.G., Kutateladze T.G. Molecular mechanism of the MORC4 ATPase activation. Nat. Commun. 2020; 11:5466. 33122719 10.1038/s41467-020-19278-8 PMC7596504

[21] Jumper J. , Evans R., Pritzel A., Green T., Figurnov M., Ronneberger O., Tunyasuvunakool K., Bates R., Žídek A., Potapenko A. et al. Highly accurate protein structure prediction with AlphaFold. Nature. 2021; 596:583–589. 34265844 10.1038/s41586-021-03819-2 PMC8371605

[22] Pastor W.A. , Stroud H., Nee K., Liu W., Pezic D., Manakov S., Lee S.A., Moissiard G., Zamudio N., Bourc’his D. et al. MORC1 represses transposable elements in the mouse male germline. Nat. Commun. 2014; 5:5795. 25503965 10.1038/ncomms6795 PMC4268658

[23] Weiser N.E. , Yang D.X., Feng S., Kalinava N., Brown K.C., Khanikar J., Freeberg M.A., Snyder M.J., Csankovszki G., Chan R.C. et al. MORC-1 integrates nuclear RNAi and transgenerational chromatin architecture to promote germline immortality. Dev. Cell. 2017; 41:408–423. 28535375 10.1016/j.devcel.2017.04.023 PMC5527976

[24] Seczynska M. , Bloor S., Cuesta S.M., Lehner P.J. Genome surveillance by HUSH-mediated silencing of intronless mobile elements. Nature. 2022; 601:440–445. 34794168 10.1038/s41586-021-04228-1 PMC8770142

[25] Desai V.P. , Chouaref J., Wu H., Pastor W.A., Kan R.L., Oey H.M., Li Z., Ho J., Vonk K.K.D., San Leon Granado D. et al. The role of MORC3 in silencing transposable elements in mouse embryonic stem cells. Epigenetics Chromatin. 2021; 14:49. 34706774 10.1186/s13072-021-00420-9 PMC8555065

[26] Dion C. , Roche S., Laberthonnière C., Broucqsault N., Mariot V., Xue S., Gurzau A.D., Nowak A., Gordon C.T., Gaillard M.-C. et al. SMCHD1 is involved in de novo methylation of the DUX4-encoding D4Z4 macrosatellite. Nucleic Acids Res. 2019; 47:2822–2839. 30698748 10.1093/nar/gkz005 PMC6451109

[27] Roubille S. , Juillard F., Escure T., Olivier B., Corpet A., Bloor S., Cohen C., Texier P., Oziol N., Haigh O. et al. The HUSH-SETDB1-MORC2 epigenetic repressor complex restricts herpesvirus infection in association with PML nuclear bodies. Proceedings of the National Academy of Sciences. 2024; 121:10.21203/rs.3.rs-2521252/v1. PMC11626126 39589886

[28] Liu N. , Lee C.H., Swigut T., Grow E., Gu B., Bassik M.C., Wysocka J. Selective silencing of euchromatic L1s revealed by genome-wide screens for L1 regulators. Nature. 2018; 553:228–232. 29211708 10.1038/nature25179 PMC5774979

[29] Yurkovetskiy L. , Guney M.H., Kim K., Goh S.L., McCauley S., Dauphin A., Diehl W.E., Luban J. Primate immunodeficiency virus proteins vpx and vpr counteract transcriptional repression of proviruses by the HUSH complex. Nat. Microbiol. 2018; 3:1354–1361. 30297740 10.1038/s41564-018-0256-x PMC6258279

[30] Zhu Y. , Wang G.Z., Cingöz O., Goff S.P. NP220 mediates silencing of unintegrated retroviral DNA. Nature. 2018; 564:278–282. 30487602 10.1038/s41586-018-0750-6 PMC6349045

[31] Lasserre A. , Marie S., Morel M., Martin M.M., Legrand A., Vauthier V., Cimarelli A., Etienne L., Margottin-Goguet F., Matkovic R. MORC2 restriction factor silences HIV proviral expression. 2023; bioRxiv doi:29 March 2023, preprint: not peer reviewed 10.1101/2023.03.29.534756.

[32] Pandiloski N. , Horvath V., Karlsson O.E., Christoforidou G., Dorazehi F., Koutounidou S., Matas J., Gerdes P., Garza R., Jönsson M.E. et al. DNA methylation governs the sensitivity of repeats to restriction by the HUSH-MORC2 corepressor. Nature Comm. 2024; 15:10.1038/s41467-024-50765-4. PMC11364546 39214989

[33] Liu Y. , Tempel W., Zhang Q., Liang X., Loppnau P., Qin S., Min J. Family-wide characterization of histone binding abilities of Human CW domain-containing proteins*. J. Biol. Chem. 2016; 291:9000–9013. 26933034 10.1074/jbc.M116.718973 PMC4861470

[34] Gradia S.D. , Ishida J.P., Tsai M.-S., Jeans C., Tainer J.A., Fuss J.O. Eichman B.F. Chapter one - MacroBac: new technologies for robust and efficient large-scale production of recombinant multiprotein complexes. Methods in Enzymology, DNA Repair Enzymes: Structure, Biophysics, and Mechanism. 2017; 592:Academic Press 1–26. 10.1016/bs.mie.2017.03.008 PMC6028233 28668116

[35] Kalderon D. , Roberts B.L., Richardson W.D., Smith A.E. A short amino acid sequence able to specify nuclear location. Cell. 1984; 39:499–509. 6096007 10.1016/0092-8674(84)90457-4

[36] Vos S.M. , Pöllmann D., Caizzi L., Hofmann K.B., Rombaut P., Zimniak T., Herzog F., Cramer P. Architecture and RNA binding of the human negative elongation factor. eLife. 2016; 5:e14981. 27282391 10.7554/eLife.14981 PMC4940160

[37] Kapust R.B. , Waugh D.S. Controlled intracellular processing of fusion proteins by TEV Protease. Protein Expression Purif. 2000; 19:312–318. 10.1006/prep.2000.1251 10873547

[38] Kramer K. , Sachsenberg T., Beckmann B.M., Qamar S., Boon K.-L., Hentze M.W., Kohlbacher O., Urlaub H. Photo-cross-linking and high-resolution mass spectrometry for assignment of RNA-binding sites in RNA-binding proteins. Nat. Methods. 2014; 11:1064–1070. 25173706 10.1038/nmeth.3092 PMC6485471

[39] Röst H.L. , Sachsenberg T., Aiche S., Bielow C., Weisser H., Aicheler F., Andreotti S., Ehrlich H.-C., Gutenbrunner P., Kenar E. et al. OpenMS: a flexible open-source software platform for mass spectrometry data analysis. Nat. Methods. 2016; 13:741–748. 27575624 10.1038/nmeth.3959

[40] Dyer P.N. , Edayathumangalam R.S., White C.L., Bao Y., Chakravarthy S., Muthurajan U.M., Luger K. Reconstitution of nucleosome core particles from recombinant histones and DNA. Methods in Enzymology, Chromatin and Chromatin Remodeling Enzymes, Part A. 2003; 375:Academic Press 23–44. 10.1016/s0076-6879(03)75002-2 14870657

[41] Farnung L. , Vos S.M., Wigge C., Cramer P. Nucleosome–Chd1 structure and implications for chromatin remodelling. Nature. 2017; 550:539–542. 29019976 10.1038/nature24046 PMC5697743

[42] Stefanovsky V.Y. , Moss T. Leblanc B.P. , Rodrigue S. The cruciform DNA mobility shift assay: a tool to study proteins that recognize bent DNA. DNA-Protein Interactions, Methods in Molecular Biology. 2015; 1334:New York, NY Springer New York 195–203. 10.1007/978-1-4939-2877-4_12 26404151

[43] Rodríguez A.C. , Stock D. Crystal structure of reverse gyrase: insights into the positive supercoiling of DNA. EMBO J. 2002; 21:418–426. 11823434 10.1093/emboj/21.3.418 PMC125824

[44] Onn I. , Koshland D. *In vitro* assembly of physiological cohesin/DNA complexes. Proc. Natl Acad. Sci. U.S.A. 2011; 108:12198–12205. 21670264 10.1073/pnas.1107504108 PMC3145678

[45] Nguyen Ba A.N. , Pogoutse A., Provart N., Moses A.M. NLStradamus: a simple hidden Markov model for nuclear localization signal prediction. BMC Bioinf. 2009; 10:202. 10.1186/1471-2105-10-202 PMC2711084 19563654

[46] Cianfrocco M.A. , Wong-Barnum M., Youn C., Wagner R., Leschziner A. COSMIC2: a science gateway for cryo-electron microscopy structure determination. In Proceedings of the Practice and Experience in Advanced Research Computing 2017 on Sustainability, Success and Impact, PEARC ’17. 2017; New York, NY, USA Association for Computing Machinery 1–5.

[47] Zhao S. , Jiang E., Chen S., Gu Y., Shangguan A.J., Lv T., Luo L., Yu Z. PiggyBac transposon vectors: the tools of the human gene encoding. Transl. Lung Cancer Res. 2016; 5:120–125. 26958506 10.3978/j.issn.2218-6751.2016.01.05 PMC4758974

[48] Gillotin S. Isolation of chromatin-bound proteins from subcellular fractions for biochemical analysis. Bio. Protoc. 2018; 8:e3035. 10.21769/BioProtoc.3035 PMC8342065 34532513

[49] Dobin A. , Davis C.A., Schlesinger F., Drenkow J., Zaleski C., Jha S., Batut P., Chaisson M., Gingeras T.R. STAR: ultrafast universal RNA-seq aligner. Bioinformatics. 2013; 29:15–21. 23104886 10.1093/bioinformatics/bts635 PMC3530905

[50] Jin Y. , Tam O.H., Paniagua E., Hammell M. TEtranscripts: a package for including transposable elements in differential expression analysis of RNA-seq datasets. Bioinformatics. 2015; 31:3593–3599. 26206304 10.1093/bioinformatics/btv422 PMC4757950

[51] Anders S. , Pyl P.T., Huber W. HTSeq—A Python framework to work with high-throughput sequencing data. Bioinformatics. 2015; 31:166–169. 25260700 10.1093/bioinformatics/btu638 PMC4287950

[52] Love M.I. , Huber W., Anders S. Moderated estimation of fold change and dispersion for RNA-seq data with DESeq2. Genome Biol. 2014; 15:550. 25516281 10.1186/s13059-014-0550-8 PMC4302049

[53] Kimura Y. , Sakai F., Nakano O., Kisaki O., Sugimoto H., Sawamura T., Sadano H., Osumi T. The newly identified Human nuclear protein NXP-2 possesses three distinct domains, the nuclear matrix-binding, RNA-binding, and coiled-coil domains*. J. Biol. Chem. 2002; 277:20611–20617. 11927593 10.1074/jbc.M201440200

[54] Gaidt M.M. , Morrow A., Fairgrieve M.R., Karr J.P., Yosef N., Vance R.E. Self-guarding of MORC3 enables virulence factor-triggered immunity. Nature. 2021; 600:138–142. 34759314 10.1038/s41586-021-04054-5 PMC9045311

[55] Lowary P.T. , Widom J. New DNA sequence rules for high affinity binding to histone octamer and sequence-directed nucleosome positioning1. J. Mol. Biol. 1998; 276:19–42. 9514715 10.1006/jmbi.1997.1494

[56] Brázda V. , Laister R.C., Jagelská E.B., Arrowsmith C. Cruciform structures are a common DNA feature important for regulating biological processes. BMC Mol. Biol. 2011; 12:33. 21816114 10.1186/1471-2199-12-33 PMC3176155

[57] Maxwell A. , Gellert M. The DNA dependence of the ATPase activity of DNA gyrase. J. Biol. Chem. 1984; 259:14472–14480. 6094559

[58] Lee D.G. , Makhov A.M., Klemm R.D., Griffith J.D., Bell S.P. Regulation of origin recognition complex conformation and ATPase activity: differential effects of single-stranded and double-stranded DNA binding. EMBO J. 2000; 19:4774–4782. 10970868 10.1093/emboj/19.17.4774 PMC302069

[59] Jacobs-Palmer E. , Hingorani M.M. The effects of nucleotides on MutS-DNA binding kinetics clarify the role of MutS ATPase activity in mismatch repair. J. Mol. Biol. 2007; 366:1087–1098. 17207499 10.1016/j.jmb.2006.11.092 PMC1941710

[60] Narlikar G.J. , Sundaramoorthy R., Owen-Hughes T. Mechanisms and functions of ATP-dependent chromatin-remodeling enzymes. Cell. 2013; 154:490–503. 23911317 10.1016/j.cell.2013.07.011 PMC3781322

[61] Ban C. , Junop M., Yang W. Transformation of MutL by ATP binding and hydrolysis: a switch in DNA mismatch repair. Cell. 1999; 97:85–97. 10199405 10.1016/s0092-8674(00)80717-5

[62] Li S. , Yen L., Pastor W.A., Johnston J.B., Du J., Shew C.J., Liu W., Ho J., Stender B., Clark A.T. et al. Mouse MORC3 is a GHKL ATPase that localizes to H3K4me3 marked chromatin. Proc. Natl Acad. Sci. U.S.A. 2016; 113:E5108–E5116. 27528681 10.1073/pnas.1609709113 PMC5024608

[63] Corbett K.D. , Berger J.M. Structural dissection of ATP turnover in the prototypical GHL ATPase TopoVI. Structure. 2005; 13:873–882. 15939019 10.1016/j.str.2005.03.013

[64] Hornbeck P.V. , Kornhauser J.M., Tkachev S., Zhang B., Skrzypek E., Murray B., Latham V., Sullivan M. PhosphoSitePlus: a comprehensive resource for investigating the structure and function of experimentally determined post-translational modifications in man and mouse. Nucleic Acids Res. 2012; 40:D261–D270. 22135298 10.1093/nar/gkr1122 PMC3245126

[65] Sánchez-Solana B. , Li D., Kumar R. Cytosolic functions of MORC2 in lipogenesis and adipogenesis. Biochim. Biophys. Acta. Mol. Cell Res. 2014; 1843:316–326. 10.1016/j.bbamcr.2013.11.012 PMC3923981 24286864

[66] Hein M.Y. , Peng D., Todorova V., McCarthy F., Kim K., Liu C., Savy L., Januel C., Baltazar-Nunez R., Bax S. et al. Global organelle profiling reveals subcellular localization and remodeling at proteome scale. 2023; bioRxiv doi:18 December 2023, preprint: not peer reviewed 10.1101/2023.12.18.572249. 39742809

[67] Wang G.-L. , Wang C.-Y., Cai X.-Z., Chen W., Wang X.-H., Li F. Identification and expression analysis of a novel CW-type zinc finger protein MORC2 in cancer cells. Anat. Rec. 2010; 293:1002–1009. 10.1002/ar.21119 20225202

[68] Zanni G. , Nardella M., Barresi S., Bellacchio E., Niceta M., Ciolfi A., Pro S., D’Arrigo S., Tartaglia M., Bertini E. *De novo* p.T362R mutation in MORC2 causes early onset cerebellar ataxia, axonal polyneuropathy and nocturnal hypoventilation. Brain. 2017; 140:e34–e34. 28402445 10.1093/brain/awx083

[69] Schottmann G. , Wagner C., Seifert F., Stenzel W., Schuelke M. *MORC2* mutation causes severe spinal muscular atrophy-phenotype, cerebellar atrophy, and diaphragmatic paralysis. Brain. 2016; 139:e70. 27794525 10.1093/brain/aww252

[70] Tan W. , Park J.V., Venugopal H., Lou J.Q., Dias P.S., Baldoni P.L., Dite T., Moon K.-W., Keenan C.R., Gurzau A.D. et al. MORC2 phosphorylation fine tunes its DNA compaction activity. 2024; bioRxiv doi:28 June 2024, preprint: not peer reviewed 10.1101/2024.06.27.600912. PMC12216690 40593625

[71] Rappsilber J. , Mann M., Ishihama Y. Protocol for micro-purification, enrichment, pre-fractionation and storage of peptides for proteomics using StageTips. Nat. Protoc. 2007; 2:1896–1906. 17703201 10.1038/nprot.2007.261

[72] Zhang Y. , Bertulat B., Tencer A.H., Ren X., Wright G.M., Black J., Cardoso M.C., Kutateladze T.G. MORC3 Forms nuclear condensates through phase separation. iScience. 2019; 17:182–189. 31284181 10.1016/j.isci.2019.06.030 PMC6614601

